# Ultrastructural analysis of zinc oxide nanospheres enhances anti-tumor efficacy against Hepatoma

**DOI:** 10.3389/fonc.2022.933750

**Published:** 2022-10-27

**Authors:** Amr Hassan, Fawziah A. Al-Salmi, Tamer M. M. Abuamara, Emadeldin R. Matar, Mohamed E. Amer, Ebrahim M. M. Fayed, Mohamed G. A. Hablas, Tahseen S. Mohammed, Haytham E. Ali, Fayez M. Abd EL-fattah, Wagih M. Abd Elhay, Mohammad A. Zoair, Aly F. Mohamed, Eman M. Sharaf, Eldessoky S. Dessoky, Fahad Alharthi, Hussam Awwadh E. Althagafi, Ahmed I. Abd El Maksoud

**Affiliations:** ^1^ Department of Bioinformatics, Genetic Engineering and Biotechnology Research Institute (GEBRI), University of Sadat City, Sadat, Egypt; ^2^ Biology Department, College of Sciences, Taif University, Taif, Saudi Arabia; ^3^ Department of Histology, Faculty of Medicine, Al-Azhar University, Cairo, Egypt; ^4^ Departments of Pathology, Faculty of Medicine, Al-Azhar University, Cairo, Egypt; ^5^ Department of Public Health and Community Medicine, Faculty of Medicine, Al-Azhar University, Cairo, Egypt; ^6^ Department of Anatomy and Embryology, Faculty of Medicine, Al-Azhar University, Cairo, Egypt; ^7^ Department of Physiology, Faculty of Medicine, Al-Azhar University, Cairo, Egypt; ^8^ Research and development department, Egyptian Organization for Biological Products and Vaccines [Holding Company for Vaccine and Sera Production (VACSERA)], Giza, Egypt; ^9^ Department of Bacteriology, Immunology, and Mycology, Animal Health Research Institute (AHRI), Shebin El Kom, Egypt; ^10^ Biology Department, Faculty of Science and Arts, Al-Baha University, Al-Mikhwah, Saudi Arabia; ^11^ Department of Industrial Biotechnology, Genetic Engineering and Biotechnology Research Institute (GEBRI), University of Sadat City, Sadat, Egypt

**Keywords:** zinc oxide nanosphere, reactive oxygen species, Zn+2 ion, G2/M transition, apoptotic morphology, P53, Bax

## Abstract

Zinc oxide nanomaterial is a potential material in the field of cancer therapy. In this study, zinc oxide nanospheres (ZnO-NS) were synthesized by Sol-gel method using yeast extract as a non-toxic bio-template and investigated their physicochemical properties through various techniques such as FTIR, XR, DLS, and TEM. Furthermore, free zinc ions released from the zinc oxide nanosphere suspended medium were evaluated by using the ICP-AS technique. Therefore, the cytotoxicity of ZnO nanospheres and released Zn ions on both HuH7 and Vero cells was studied using the MTT assay. The data demonstrated that the effectiveness of ZnO nanospheres on HuH7 was better than free Zn ions. Similarly, ZnO-Ns were significantly more toxic to HuH7 cell lines than Vero cells in a concentration-dependent manner. The cell cycle of ZnO-Ns against Huh7 and Vero cell lines was arrested at G_2_/M. Also, the apoptosis assay using Annexin-V/PI showed that apoptosis of HuH7 and Vero cell lines by ZnO nanospheres was concentration and time-dependent. Caspase 3 assay results showed that the apoptosis mechanism may be intrinsic and extrinsic pathways. The mechanism of apoptosis was determined by applying the RT-PCR technique. The results revealed significantly up-regulated Bax, P53, and Cytochrome C, while the Bcl_2_ results displayed significant down-regulation and the western blot data confirmed the RT-PCR data. There is oxidative stress of the ZnO nanospheres and free Zn^+2^ ions. Results indicated that the ZnO nanospheres and free Zn^+2^ ions induced oxidative stress through increasing reactive oxygen species (ROS) and lipid peroxidation. The morphology of the HuH7 cell line after exposure to ZnO nanospheres at different time intervals revealed the presence of the chromatin condensation of the nuclear periphery fragmentation. Interestingly, the appearance of canonical ultrastructure features of apoptotic morphology of Huh7, Furthermore, many vacuoles existed in the cytoplasm, the majority of which were lipid droplets, which were like foamy cells. Also, there are vesicles intact with membranes that are recognized as swollen mitochondria.

## Introduction

Hepatocellular carcinoma is an aggressive malignancy and is ranked as the third most common cause of cancer-related death globally (1). Annually, more than 600,000 people die due to liver cancer (2). Generally, hepatocellular carcinoma (HCC) is diagnosed at end-stage after metastasizing (3). Hepatocellular carcinoma (HCC) is defined as the progressive development of pre-neoplastic and neoplastic lesions and the acquisition of multiple genetic and epigenetic events contributing to the biochemical and molecular heterogeneity of the disease (4, 5). Hepatic cancer is widely spread in Asian countries because of the increasing number of patients suffering from chronic viral hepatitis. HuH7 is a hepatocyte cell line established in 1982 from a 57-year-old Japanese male with well-differentiated hepatocellular carcinoma (6). It’s available in the Japan Health Science Research Resources Bank (catalog number JCRB0403) (7). Currently, there are many strategies for HCC therapy, like surgery, radiotherapy, or chemotherapy. Unfortunately, HCC is more resistant to chemotherapy (8–11). Sorafenib is the only drug that is effective against HCC (12). Recently, there has been progress in the treatment of HCC. Only 14% of hepatic cancer patients survived after treatment (13). Chemotherapy is inactive in clinical trials against hepatic cancer due to its adverse effects (14). Therefore, modern therapeutic agents advance with better efficiency against HCC such as Atezolizumab and Beacizumab (15). Scientists are now working on allowing malignant cells to undergo apoptosis after identifying biochemical and morphological markers of apoptotic cells (16, 17). P53 is a tumor suppressor protein that has a powerful caretaker to protect cells from malignant transformation by transcriptional up-regulation of pro-apoptotic DNA repair and cell cycle arrest-related proteins (18). Therefore, nanobiotechnology is considered the preferable approach to identify a novel, sophisticated therapy (19). In the last decade, nanomaterials have played a central role in medical applications such as tumor diagnosis and therapy. It can be classified as a noble metal or a metal oxide material. Also, it is classified into one dimension and two dimensions (19). Nanomedicine is a novel field have utilize the path for novel targeted cancer therapies by allowing therapeutic compounds to be encapsulated in nanoparticulate materials and delivered selectively to tumors *via* passive permeation and active internalization mechanisms. Employing nanoparticles for therapeutic purposes has also been found to minimize resistance, addressing one of the most significant obstacles to conventional therapy (20). Metal oxide nanoparticle such as ZnO NPs and Fe_3_O_4_ NPs have shown a promising anticancer behaviour besides its therapeutic activity against other diseases such as diabetes (20), microbial infections (21), inflammations (22), and wound healing (17) and environmental (23). ZnO-NPs have received considerable attention in various fields due to their excellent physicochemical properties, safety, biodegradability (24), and their fast delivery to different tissues and organs in addition to various biological purposes including drug delivery and immune-modulatory agent (Kalpana et al., 2018 (25, 26);). Zinc oxide nanoparticles have been applied in biomedical and preclinical research especially in cellular imaging and drug delivery. Fujihara and colleagues reported that intravenous administration of ZnO nanoparticles can be accumulate in several tissues, particularly lung tissues, and elicit ROS-related phenomena using healthy mice (27, 28). In addition, ZnO-NPs can also be approved to have a potential molecular effect including a reduction in cellular viability, loss of membrane integrity, and activation of the programmed cell death (apoptosis) (29). Also, zinc oxide is approved by the US Food and Drug Administration (FDA) for its properties like stability, safety, and the intrinsic potential to neutralize UV radiation. Based upon the unique properties of zinc oxide nanoparticles, here, zinc oxide nanospheres were synthesized by a green chemistry technology using yeast as a bio-template. Then, the cytotoxicity, apoptotic mechanisms, and antioxidant biomarkers of zinc oxide nanospheres were determined against Hepatoma HuH7 and green kidney monkey cell lines (Vero). Furthermore, the ultrastructure analysis of HuH7 cells was carried out to observe apoptosis stages in the human hepatotome.

## Materials and methods

### Preparation of ZnO nanosphere

Zinc oxide nanospheres were synthesized by using a modified sol-gel method (30). Briefly, three grams of yeast extract were nurtured in 100 mL of ultrapure water and left for one hour. Then, 25 mM of zinc acetate solution were added and mixed it with 50 ml of yeast extract solution under vigorous stirring at 1400 rpm for one hour, followed by thermal treatment at 500 °C for two hour. The resulting white precipitate has been dried and powdered and is ready to be characterized.

### Characterization of ZnO nanosphere

ZnO nanospheres spectra were assayed by ultraviolet-visible (UV-VIS) spectrometry (JASCO V-630 spectrophotometer, Japan). A Fourier transformed infrared (FT-IR) spectrum of the ZnO nanospheres was characterized *via* the Nicolet 6700 apparatus (Thermo Scientific Inc., USA). The crystalline nature and grain size were analyzed by X-ray powder diffraction (XRD) at a temperature of 25–28 °C using a D8 Advance X-ray diffractometer (Bruker, Germany) with a nickel (Ni) filter and CuKα (λ= 1.54184 A^0^) radiation as an X-ray source. The average size of ZnO nanospheres in cell culture medium was determined by dynamic light scattering (DLS) (Nano-ZetaSizer-HT, Malvern Instruments, Malvern, UK) (30). The morphology of zinc nano-spheres was examined by Field Emission Transmission Electron Microscopy (FETEM) (JSM 2100F, Joel Inc., Tokyo, Japan) at 15 and 200 kV accelerator voltages, respectively.

### Measurement of Zn (II) released from ZnO nanospheres

Following quantification of the final concentration of released zinc ions from suspended ZnO-NS, the following products occurred: Firstly, dilution the stock suspension of a concentration of 100 μg/ml ZnO nanospheres by Dulbecco’s Modified Eagle’s Medium (DMEM) to a final volume of 15 ml. Then, incubate all samples at 37 C in a humidified atmosphere (with 5% CO_2_) at different times (0, 3, 6, 18, and 24 h). followed by centrifugation for 20 min at 10,000 x g, and then transferring the supernatant (10 ml) into a test tube containing 0.5 ml of Conc HNO_3_. The solution was filled with up to 50 ml of water and the Zn^+2^ ions were quantified with inductively coupled plasma atomic emission spectroscopy (ICP-AES) (Perkin-Elmer, USA) (31).

### Cell lines

Human hepatocellular carcinoma (HuH7) cells (catalog number JCRB0403) were obtained from the Japan Health Science-Research Resources Bank. The Green Kidney Monkey (Vero cells) was purchased from ATCC (American Type Culture Collection) (Clone CCL-81). The cells were maintained in a 95% air and 5% CO_2_ humidified atmosphere at 37°C. DMEM and MEM-E medium supplemented with 10% FBS and 1% PS were used for routine sub-culturing and all experiments.

### Cell viability assays

The viability of HuH7 and Vero cell lines was assessed by the MTT assay as described by Mossman (32) with some modifications. Briefly, 1 × 10^4^ cells/well were seeded in 96-well plates and exposed to Zinc Oxide Nanosphere and ZnCl_2_ at the concentrations of 100μg/ml, 50μg/mL, 25μg/mL, 12.5μg/mL and 6.25 μg/mL for 24 hour. At the end of exposure, culture medium was removed from each well to avoid interference of ZnO NPs and ZnCl_2_, then replaced with new medium containing MTT solution (0.5 mg/mL) in an amount equal to 10% of the culture volume and incubated for 4 hour at 37°C until a purple-colored formazan product developed. The resulting formazan product was dissolved in acidified isopropanol. Further, the 96-well plate was centrifuged at 2300 × g for 5 minutes to settle the remaining ZnO NPs and ZnCl_2_. Then, a 100 μL supernatant was transferred to the other fresh wells of a 96-well plate, and absorbance was measured at 570 nm by a microplate reader (ELX-800 n, Biotek, USA).

### DNA content analysis

HuH7 cells (1 × 10^6^) were seeded in T25 flasks and at 70–80% con-fluence the cells were treated with suspended ZnO nanospheres at different concentrations. After 24 h, media was aspirated and cells were harvested with 0.25% trypsin and fixed in70% ice cold ethanol at −20^°^C for 30 min. Cell pellets were washed with PBS and re-suspended in 500 µl PBS containing 20 µl RNAse (5 mg/ml) and stained with 10 µl PI (1 mg/ml) for 30 min at 37^°^C. Data acquisition was performed with a fluorescence-activated cell sorter (FACS Canto, Becton-Dickinson, Franklin Lakes, NJ). For each sample, 10,000 events were acquired and the analysis was carried out using BD FACS Diva software (Becton-Dickinson, Franklin Lakes,NJ-USA) (33).

### Apoptosis assay with Annexin V-FITC/PI staining

Huh7 and Vero cells (1x10^4^/well) were seeded in T25 flasks and treated different concentrations of ZnO nanospheres at different times. Cells were then harvested and washed twice with PBS, resuspended in 0.5 ml binding buffer containing FITC-Annexin V and PI and kept at room temperature in the dark for 30 min. The fluorescence of the cells was then analyzed by flow cytometer (Becton-Dickinson, Franklin Lakes, NJ -USA). For each sample, 10,000 events were acquired and the analysis was carried out using BD FACS Diva software (Becton-Dickinson, Franklin Lakes, NJ-USA) (33).

### Cell apoptotic mechanisms for RNA extraction and quantitative RT-PCR

The HuH7 cells were cultured in six-well plates and exposed to ZnO nanospheres (100 μg/ml) for 24 hour. At the end of the exposure process, according to the manufacturer’s protocol, total RNA was extracted by RNeasy mini Kit (Qiagen,Valencia, CA, USA) according to the manufacturer’s instructions. Concentration of the extracted RNA was determined using Nanodrop 8000 spectrophotometer (Thermo-Scientific, Wilmington, DE), and the integrity of RNA was visualized on a 1% agarose gel using a gel documentation system (Universal Hood II, BioRad, Hercules, CA). The first strand of cDNA was synthesized from 1 µg of total RNA by reverse transcriptase using M-MLV (Promega, Madison, WI) and oligo (dT) primers (Promega) according to the manufacturer’s protocol. Quantitative real-time PCR was performed by QuantiTect SYBR Green PCR kit (Qiagen) using an ABI PRISM 7900HT Sequence Detection System (Applied Biosystems, Foster City, CA). Two microliters of template cDNA were added to the final volume of 20 µL of reaction mixture. Real-time PCR cycle parameters included 10 minutes at 95°C followed by 40 cycles involving denaturation at 95°C for 15 seconds, annealing at 60°C for 20 seconds, and elongation at 72°C for 20 seconds. The sequences of the specific sets of primer for p53, bax, bcl-2, cytochrome C, and β-actin. Expressions of selected genes were normalized to the β-actin gene, which was used as an internal housekeeping control. All the real-time PCR experiments were performed in triplicate, and data were expressed as the mean of at least three independent experiments (34–36).

### Gene expression by flow cytometry

All flow cytometric analyses were performed on a FACS Calibur flow cytometer (BD, Biosciences, CA, USA). The instrument was aligned and calibrated daily with the use of a 4-color mixture of CaliBRITE beads (BD, Biosciences) and FACS Comp Software (BD, Biosciences), according to the manufacturer’s instructions (35). The flow cytometry technique evaluated the Bcl_2_, Bax, P53, and Cytochrome C oncoproteins. After HuH7 cells were treated with ZnO nanospheres (100 μg/ml) for 24 h. Cells were collected by cold centrifugation at approximately 5000 x g for 10 min, then washed twice and re-suspended in 500 µl of cold (+4 °C) 1X PBS buffer containing Triton X-100 (permeabilization step). After centrifugation as previously described, the supernatant was removed, and the pellet was re-suspended again in PBS containing 1% BSA and diluted primary antibody rabbit monoclonal antibody (1:100) (Oncogene, Cambridge, MA, USA) for P53, Bax, Bcl_2_, and cytochrome C, followed by incubation at room temperature for 1 hr. After centrifugation, the pellet was washed three times using PBS, and the cells were incubated with secondary antibodies, anti-rabbit (all from Santa Cruz Biotechnology, USA) in a dilution of 1:100, followed by incubation in the dark for 30 min at RT. Finally, the cells were centrifuged, and the supernatant was removed. The cells had been washed as previously described. The pellet was finally re-suspended in 500 µl PBS. The cells were immediately analyzed by flow cytometry (BD FASC Calibur-USA) (37).

### Western analysis

After treatment with various concentrations of ZnO-NPs, cells were collected by centrifuging at 100 x g and washed twice with ice-cold PBS (pH 7.4) and lysed in 1 ml lysis buffer (2 mM Tris-HCl, pH 8.0, 1% Nonident P-40, 13.7 mM NaCl, 10% glycerol, 1 mM sodium orthovanadate (NaVO3), 1 mM phenylmethyl-sulfonyl fluoride and 10 µg/ml Aprotinin) for 20 min on ice. Lysates were centrifuged at19 000 x g for 15 min at 4°C, and aliquots of the supernatants were used to determine protein concentration using the BCA assay (Pierce, Rockford, USA). Aliquots containing equal amounts of proteins (20 - 30 µg) were boiled in 2 x sodium dodecyl sulfate (SDS) sample loading buffer (125 mM Tris-HCl, 4% (w/v) SDS, 20% (v/v) glycerol, 10% (v/v) 2-mercaptoethanol, pH 6.8) before being resolved on a 12% sodium dodecyl sulfate-polyacrylamide gel (SDS-PAGE). Proteins on the gels were then electro-blotted onto Immobilon-P transfer membrane (Millipore Corporation, Bedford, USA) using a blotting buffer (10% methanol, 10 mM CAPS, pH 11.0) at 200 mA for 2 h at 4°C. Following electro-blotting, the membranes were blocked with 0.05% TBS-Tween (20 mM Tris-HCl, 200 mM NaCl, pH 7.4) containing 5% non-fat dry milk for 1 h at room temperature. After blocking, the membranes were washed three times for 10 min each with wash buffer (0.05% TBS-Tween without milk), and then incubated with specific primary goat anti mouse Bcl-2 antibody (1: 1000), goat anti-mouse Bax antibody (1: 500), goat anti-mouse p53 antibody (1: 1000) and goat anti-mouse anti- cytochrome c (1:2000) for overnight at 4°C. After washing three times with washing buffer for 10 min each, membranes were further incubated for 1 h in the presence of a peroxidase (HRP)-conjugated secondary antibody (1: 10 000) diluted with blocking buffer. The membranes were washed again as described above and immunoreactive proteins were then detected using the Western blotting luminol reagent (Santa-Cruz Biotechnology Inc., Santa-Cruz, CA, USA) following the manufacturer’s protocol (38).

### Caspase-3 assay

The Activity of caspase-3 enzyme was determined by the standard fluorometric microplate assay (33). Briefly, HuH7 cells (1×10^4^ cells/well) were cultured in a 96-well plate and exposed to ZnO nanospheres at concentrations of 50, 100, 150, and 200 μg/mL for 24 hour. After the exposure was completed, the cells were harvested in ice-cold phosphate buffer saline for preparing the cell lysate. Further, a reaction mixture containing 30 μL of cell lysate, 20 μL of (Ac-DEVD-pNA) (caspase-3 substrate), and 150 μL of protease reaction buffer (50 mM Hepes, 1 mM EDTA, and 1 mM DTT) (pH 7.2) was incubated for 15 min. Fluorescence of the reaction mixture was measured at 5-minute intervals for 15 minutes at excitation and emission wavelengths of 430/535 nm using an ELISA reader apparatus (ELX-800n, Biotek, USA). A 7-amido-4-tri-fluoromethyl coumarin (AFC) standard ranging from 5m to 15 μM was prepared, and its fluorescence was recorded to calculate caspase-3 activity in terms of pmol AFC released/minute/mg protein.

### Oxidative stress biomarkers

#### ROS measurement

ROS was measured using 2, 7-dichlorofluorescein diacetate (DCFH-DA). The DCFHDA passively enters the cell, where it reacts with ROS to form the highly fluorescent compound dichlorofluorescein (DCF). In brief, 10 mM DCFH-DA stock solution (in methanol) was diluted in culture medium without serum or another additive to yield a 100 μM working solution. HuH7 cells were treated with ZnO NPs at a concentration of 100 μg/mL for 24 hour. At the end of exposure, cells were washed twice with HBSS and then incubated in 1 mL of working solution of DCFH-DA at 37°C for 30 minutes. Cells were lysed in an alkaline solution and centrifuged at 2300 × g for 10 minutes. A 200 μL supernatant was transferred to a 96-well plate, and fluorescence was measured at 485 nm excitation and 520 nm emissions using a microplate reader (ELX-800n, Biotek, USA). The values were expressed as a percent of fluorescence intensity relative to control wells (33).

#### Lipid peroxidation measurement

HuH7 cells were exposed to different ZnO nanospheres and free Zn^+2^ ions (released from 100 μg/ml ZnO-nanospheres) for 24 h. After the exposure, the cells were washed and harvested in cold PBS at 4°C. The harvested cell pellets were lysed using a cell lysis buffer (20 mM Tris-HCl [pH 7.5], 150 mM NaCl, 1 mM Na_2_EDTA, 1% Triton X 100, and 2.5-mM sodium pyrophosphate). After cold centrifugation at 15,000 g for 10 min, the supernatant (cell extract) was maintained on ice until assayed for oxidative-stress biomarkers. The extent of membrane lipid peroxidation (LPO) was estimated by quantifying malondialdehyde (MDA) (39). MDA is one of the final products of membrane LPO. In brief, 0.1 ml of cell extract was mixed with 1.9 ml of 0.1 M sodium phosphate buffer (pH 7.4) and incubated at a temperature of 37°C for 1 hour. After precipitation with trichloroacetic acid (TCA) (5% v/v), the incubated mixture was centrifuged (2500 g for 15 min at room temperature). The supernatant was collected and to which 1 ml of TBA (1% v/v) was added and placed in boiling water for 15 min. After cooling to room temperature, the absorbance of the mixture was taken at a wavelength of 532 nm and converted to nmol/mg protein using the molar extinction coefficient of 1.56×10^5^ M^-1^ Cm^-1^ (40).

### Antioxidant biomarkers

#### Assay for reduced glutathione (GSH)

The level of the reduced glutathione (GSH) was estimated using Ellman’s reagent (41). Based on the development of yellow color when DTNB (5, 5’ dithiobis-(2-nitrobenzoic acid) is added to compounds containing sulfhydryl groups. In brief, mixed with 100 µg of ZnO nanospheres and Zn^+2^ ions (released from 100 µg of ZnO nanospheres) after treatment with Huh7 cell line for 24 hr. was added to 0.3 ml of 0.25% sulfosalycylic acid, and then tubes were centrifuged at 2500 ×g for 15 min. Supernatant (0.5 ml) was mixed with 0.025ml of 0.01 M DTNB and 1 ml phosphate buffer (0.1 M, pH = 7.4). The absorbance at 412 nm was recorded. Finally, total GSH content was expressed as nmol GSH/mg protein.

#### Assay for nitric oxide (NO)

Nitric oxide (NO) radical scavenging assay was carried out oxide radical scavenging activity was carried out as per the method of Green et al. (1982) (42). NO radicals were generated from sodium nitroprusside solution. 0.6 mL of 10 mM sodium nitroprusside was mixed with 100 µg of ZnO nanospheres and Zn ^+2^ ions (released from 100 µg of ZnO nanospheres) after exposure to Huh7 cell line for 24 hr. The mixture was incubated at 25°C for 150 min, followed by mixing with 1.0 mL of pre-prepared Griess reagent (1% sulfanilamide, 0.1% naphthyl ethylenediamine dichloride, and 2% phosphoric acid). Ascorbic acid and trolox were used as standards. The absorbance was measured at 546 nm. The inhibition was calculated by the following equation:


$$\%\;Inhibition\;of\;NO\;radical\;=\;\left[A_{0}-A_{1}\right]/A_{0}\times \;100$$

Where, *A*_0_ is the absorbance before the reaction and *A*_1_ is the absorbance after the reaction has taken place with Griess reagent. The decreasing absorbance indicates a high NO scavenging activity.

#### Assay for superoxide dismutase (SOD)

Superoxide dismutase (SOD) assay is a mixture containing sodium pyrophosphate buffer, nitro blue tetrazolium (NBT), phenazine methosulphate (PMS), reduced nicotinamide adenine dinucleotide (NADH), and the required volume of cell extract. Superoxide dismutase enzyme activity is defined as the amount of enzyme required to inhibit chromogen production (optical density at 560 nm) by 50% in 1 minute under assay conditions and is expressed as specific activity in units/mg protein (33).

### Ultrastructure of HuH7 using a transmission electron microscope

HuH7 cells were remedied with ZnO nanospheres at a concentration of 100 μg/ml at different time intervals. After the exposure, cells were collected and washed with PBS, then fixed in ice-cold glutaraldehyde (2.5%) for one hour. The cells were washed with PBS three times for 15 min and post-fixed in OsO_4_ (1%) for one hour, then stained with uranyl acetate (2%) for 30 min at room temperature. The cells were dehydrated by serial dilutions of ethanol (50, 70, and 90%) for 15 minutes each, followed by 20 minutes in ethanol (100%) and 20 minutes in acetone (100%), respectively. Consequently, the cells were embedded in Epon812. Ultrathin sections (120 nm) were obtained and stained with uranyl acetate (2%) for 20 min, and lead citrate for 5 min, then examined using Field Emission Transmission Electron Microscopy (FETEM) (JSM 2100F, Joel Inc., Tokyo, Japan) at accelerating voltages of 15 kV and 200 kV, respectively (43).

### Statistical analysis

Statistical analysis was expressed as the mean ± standard deviation of triplicate (independent) experiments. Two sample comparisons of means were carried out using Student’s t-test analysis. All analyses were conducted using SPSS 17.0 software (SPSS Inc., Chicago, IL, USA). P <0.05 was considered a statistically significant difference.

## Result and discussion

### Characterization of ZnO nanospheres

**Figure 1:**
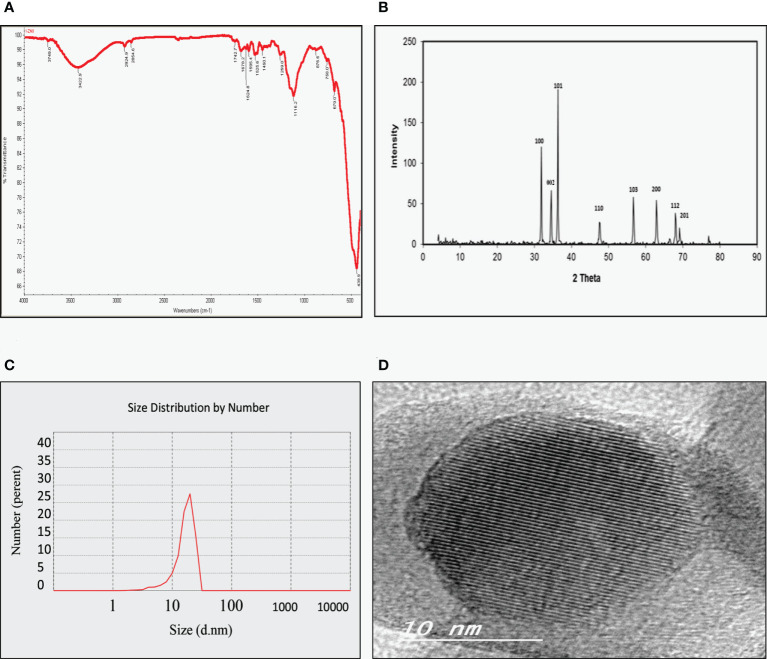
Physicochemical characterization of Zinc Oxide nanospheres. **(A)** FT-IR spectra **(B)** XRD patterns of ZnO nanospheres **(C)** DLS of ZnO nanospheres **(D)** HR-TEM of ZnO nanospheres.

As shown in **Figure 1A**, the FT-IR spectra of the ZnO nanospheres, there is a band at 3,429 cm^-1^ corresponding to the hydroxyl group of a water molecule on the surface of ZnO nanospheres that occurred because of the ZnO-nanosphere’s thermal treatment at 500°C. The band at 1,628 cm^-1^ is related to the OH bend in ZnO. Also, a strong band at 418 cm-1 was attributed to ZnO. As illustrated in **Figure 1B**, the XRD patterns of ZnO nanospheres showed that the peaks at 2 θ = 31.746°, 34.395°, 47.526°, 56.549°, 62.832°, 67.893° and 69.028° were assigned to (100), (002), (101), (110), (103), (200), (112), and (201) of ZnO nanospheres. All peaks are consistent with a polycrystalline Wurtzite structure (Zincite, JCPDS no. 89-1397). There are no characteristic peaks of any impurities, and this indicates a high ZnO nanospheres quality. Scherer’s equation estimated the average crystallite size (d) of ZnO nanospheres to be approximately 20 nm. DLS determined the average hydrodynamic size of the ZnO nanospheres in cell culture media, and it was about 20 nm, as revealed in **Figure 1C**.


d = kλβ Cos θ

As **Figure 1D** showed, TEM images confirmed that the morphological shape of the nanosphere with approximately size of 20 nm. Also, it was like the data obtained by XRD.

### Measurement of Zn (II) released from ZnO nanospheres

**Figure 2:**
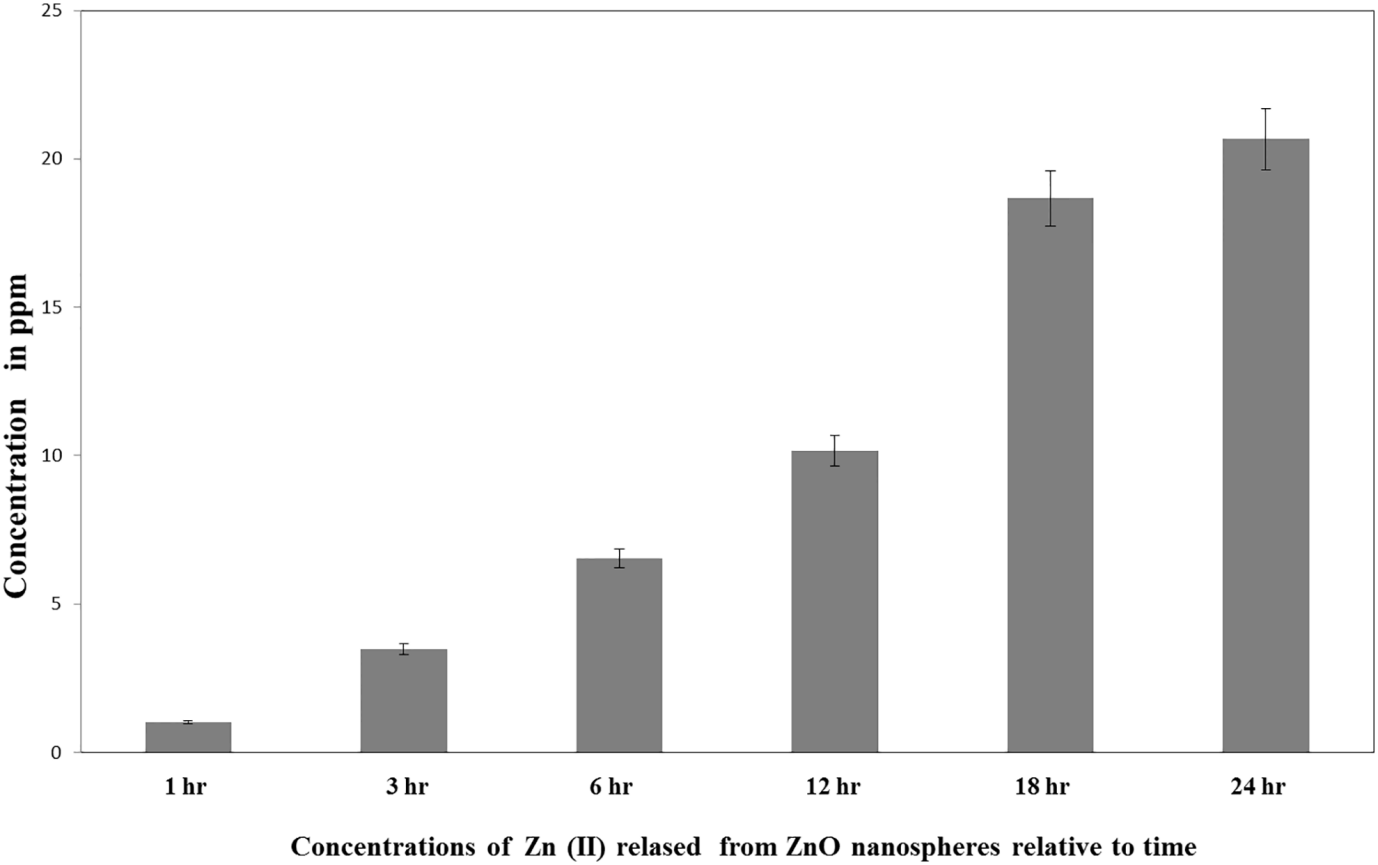
Released Zn^+2^ ions from ZnO nanospheres by ICP-AES.

ICP-AES measured the quantity of the Zn (II) ions released in the supernatant of the dispersed ZnO nanospheres (100 μg/ml). As presented in **Figure 2**, the total amount of ZnO nanospheres varied within different intervals of time. Free Zn^+2^ ions were 20ppm after 24 h, 15 ppm after 18 h, 10 ppm after 12 hr, 6.5 ppm after 6 h, 3.5 ppm after 3 h, and 1.0 ppm after 1 hr.

### Cell viability by MTT assay

**Figure 3:**
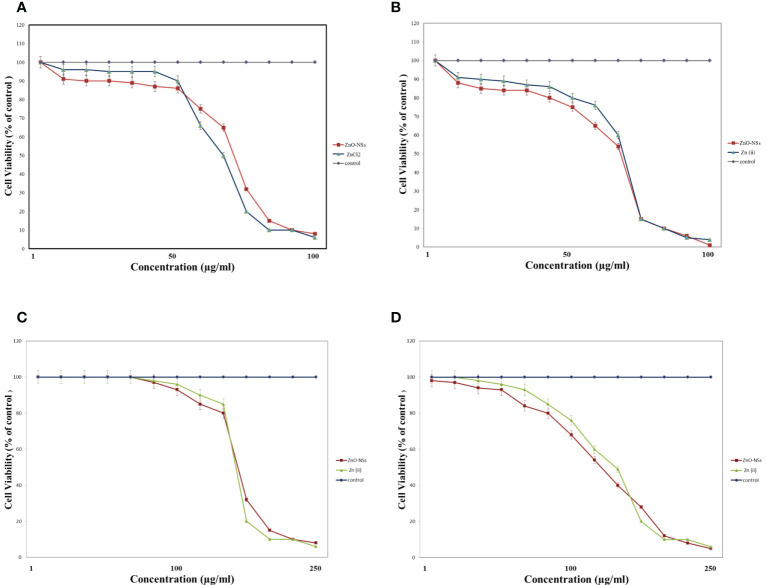
Cytotoxicity effect of ZnO nanospheres and released Zn ion different concentrations on HuH7 cell line with concentrations using MTT assay. **(A)** Cytotoxicity effect of ZnO nanospheres and released Zn ion different concentrations on HuH7 cell lines with concentrations using MTT assay after 24 hour. **(B)** Cytotoxicity effect of ZnO nanospheres and released Zn ion different concentrations on HuH7 cell line with concentrations using MTT assay after 48 hour. **(C)** Cytotoxicity effect of ZnO nanospheres and released Zn ion different concentrations on Vero cell line with concentrations using MTT assay after 24 hour. **(D)** Cytotoxicity effect of ZnO nanospheres and released Zn ion different concentrations on Vero cell line with concentrations using MTT assay after 48 hour.

MTT assay is the best technique to measure the cytotoxicity of ZnO nanospheres and ZnCl_2_ against human hepatocellular carcinoma (HuH7) and green kidney monkey cell lines (Vero). As shown in **Figure 3A**, the viability of HuH7 cells was reduced from 100% at 0.5 µg to less than 10% and 15% (for ZnO–Ns and ZnCl2, respectively) after being treated with 100 µg (ZnO nanospheres and ZnCl_2_) for 24 hour. Similarly, as shown in **Figure 3B**, the viability of HuH7 cells after 48 hour of treatment was reduced from 100% to 20% and 15% for ZnO–Ns and ZnCl_2_, respectively.

The viability of Vero cells was less affected than that of HuH7. As **Figure 3C** displays, after treatment of Vero cells by ZnO nanospheres and ZnCl_2_, for 24 hour, the viability of Vero cells decreased from 100 percent at 0.25 µg to less than 15 and 10 percent at 250 µg of ZnO nanospheres and ZnCl_2_, respectively. Similarly, the viability of Vero cells after 48 hour of exposure to ZnO-Ns and ZnCl_2_ was reduced from 100 percent to 20 and 15 percent. As **Figure 3D** shows, the results changed gradually after exposure of Vero cells to ZnO nanospheres and ZnCl_2_ for 48 hour.

### DNA content analysis

**Figure 4:**
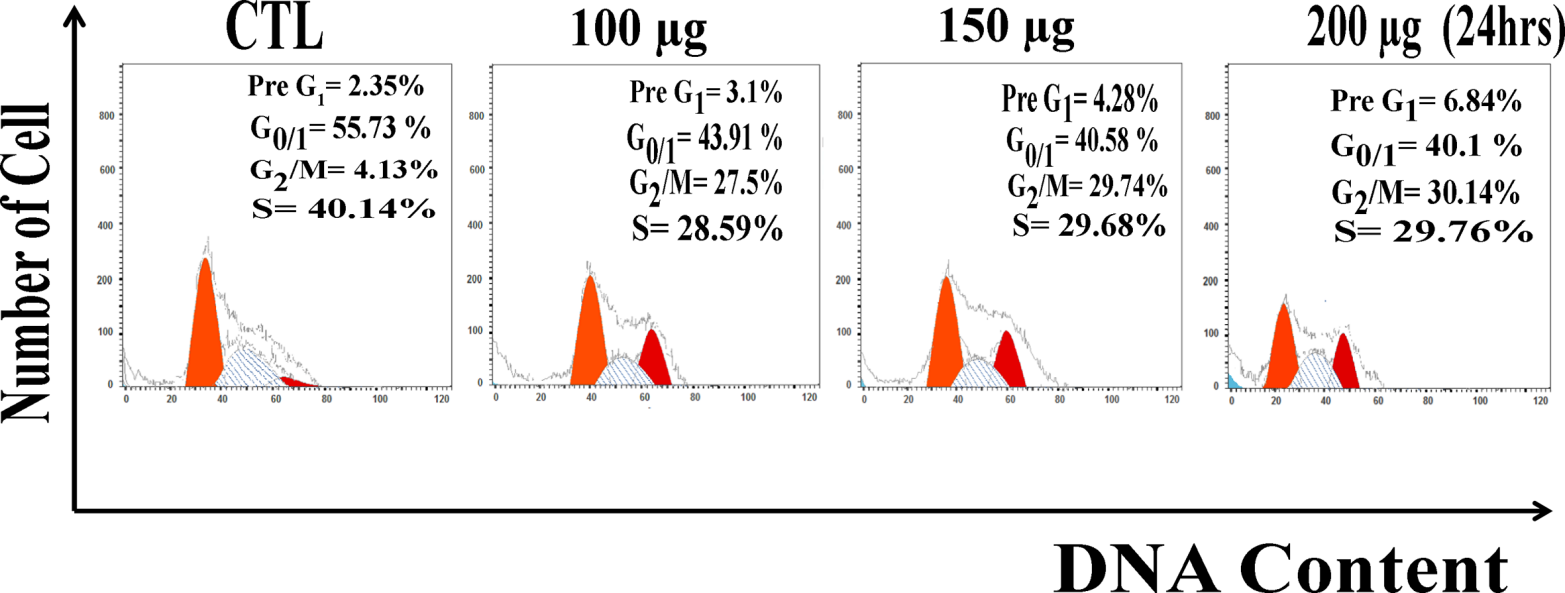
DNA content analysis HuH7 cell line treated with of ZnO nanospheres.

Due to extensive ROS generation and DNA damage, cells may arrest at different phases of the cell cycle. After 24 h treatment Huh7 were analyzed for the distribution of cell cycle phases. Measurement of the cell cycle phase ratio using flow cytometry with propidium iodide (PI) staining to investigate the anti-proliferative effect of zinc oxide nanospheres was triggered by cell cycle arrest. As shown in **Figure 4**, ZnO nanospheres treatment enhanced the accumulation of the HuH7 cells at the G_2_/M phase significantly (P<0.05) compared with the control. The percentage of the cells at G_2_/M increased significantly (P<0.05) with increasing ZnO nanospheres concentration. The growth of the HuH7 treated with 200 µg/ml ZnO nanospheres was about 55.73, 4.13, and 40.14% at G_0_/1, G_2_/M, and S, respectively, compared to the non-treated cells, in which the growth was about 40.1, 30.4, and 29.6% at G_0_/1, G_2_/M, and S, respectively, which indicated that the cell cycle arrest at G_2_/m phase is activated by Cyclin-A (CDK1).

### Huh7 cells are induced to apoptosis by zinc oxide nanospheres

**Figure 5:**
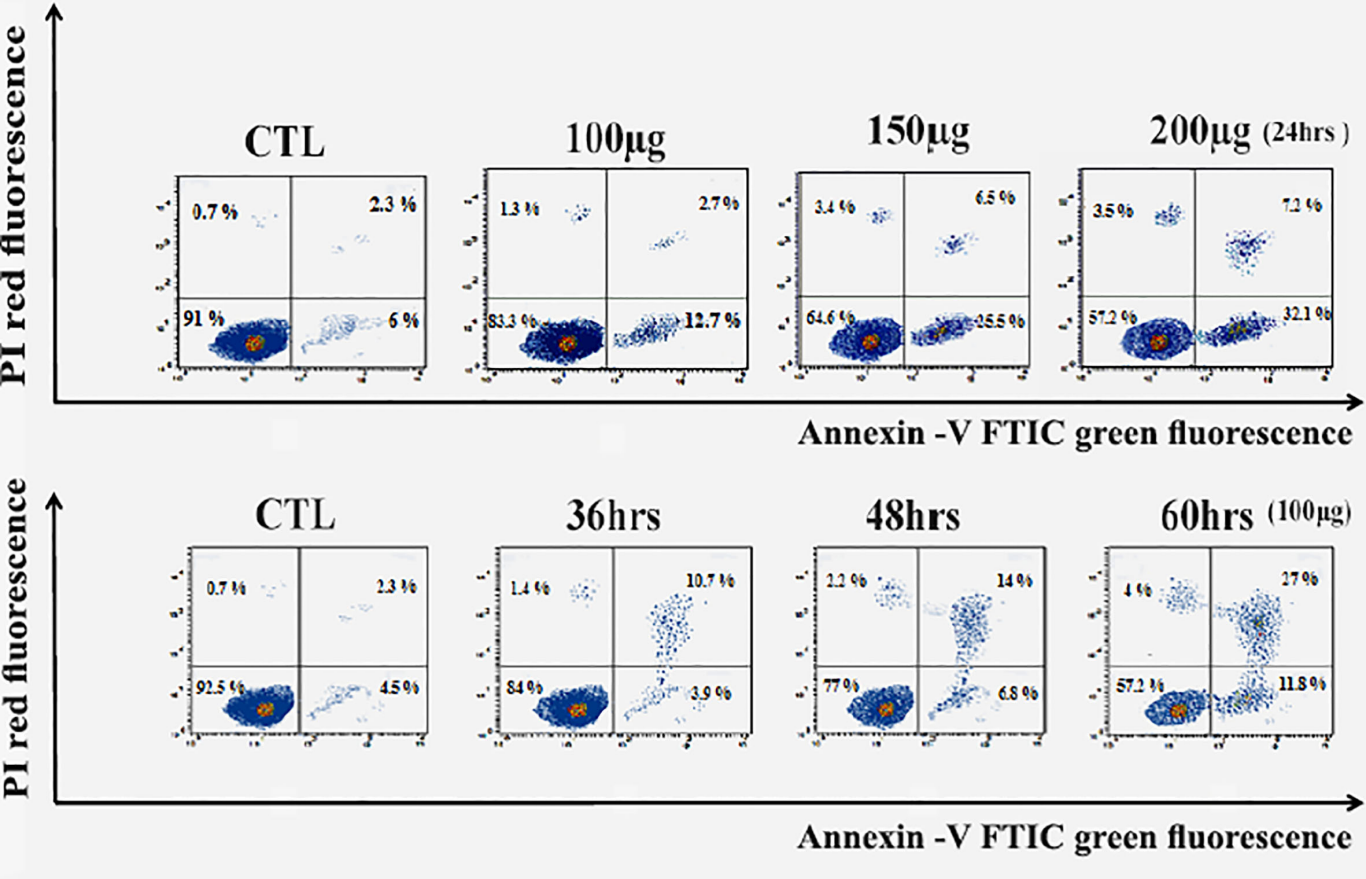
Apoptosis Assay with Annexin V-FITC/PI staining of HuH7 cell line. LL, Viable cells (Annexin V −/PI −), LR, early apoptotic cells (Annexin V +/PI −), UL, late necrotic cells (Annexin V −/PI +) and UR, late apoptotic/necrotic cells (Annexin V +/PI +).

As a result, **Figure 5** revealed a flow cytometric analysis of zinc oxide nanospheres-induced apoptosis in Huh7 cells using Annexin V-FITC/PI staining. The percentage of the apoptotic cells (including early and late apoptotic cells) increased with the increasing concentration of ZnO nanospheres from 8.3 to 39.3%, respectively. Also, as the time of treatment increased, the apoptotic percentage increased from 6.8 to 38.8%.

**Figure 6:**
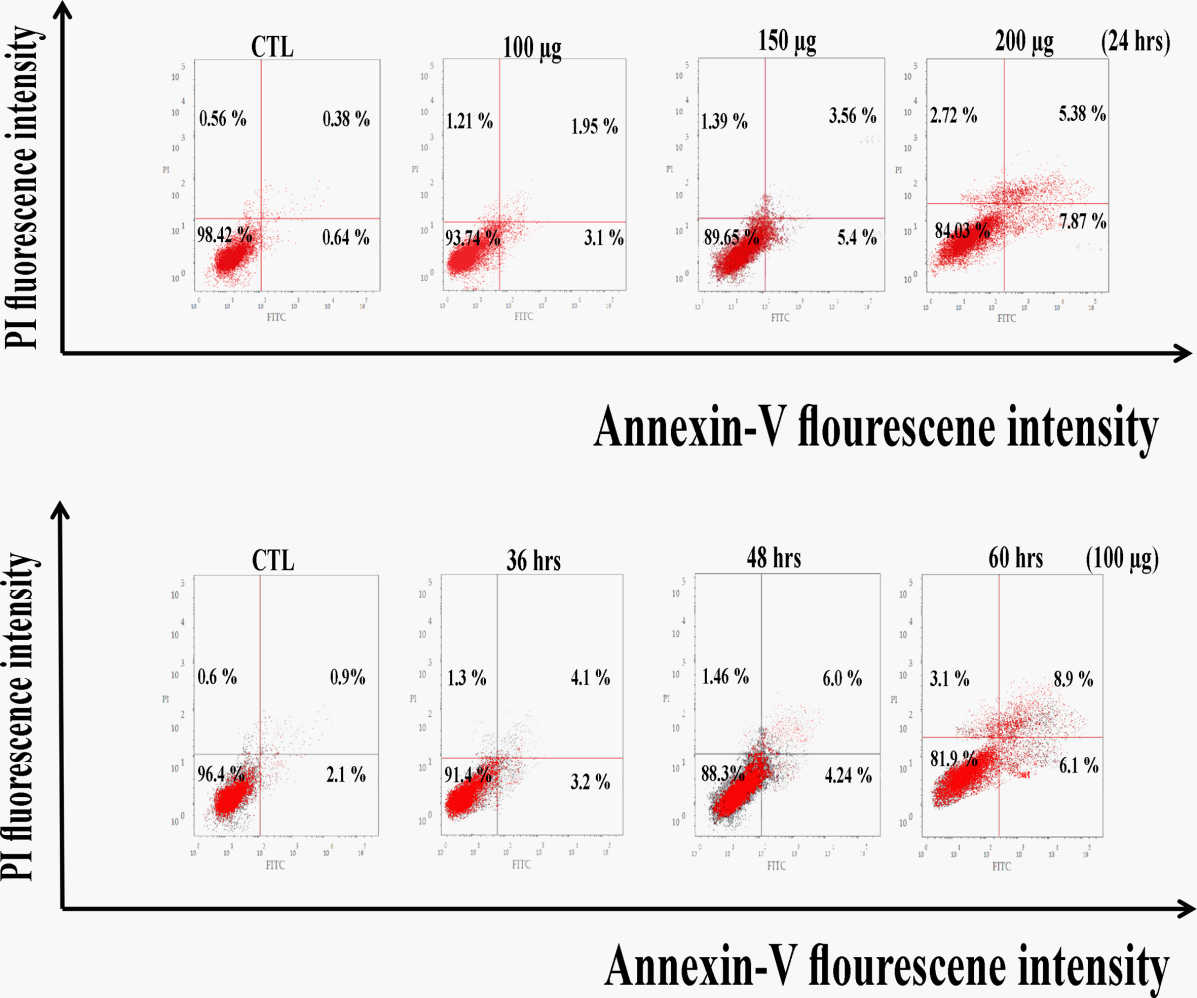
Apoptosis Assay with Annexin V-FITC/PI staining of Vero cell line.

Also, zinc oxide nanosphere has less effect on Vero cells than on HuH7. As **Figure 6** displayed, the percentage of apoptotic cells (including early and late apoptotic cells) increased with the increasing concentration of ZnO nanospheres from 1.02 to 13.25%, respectively. Also, as the time of exposure increased, the apoptotic percentage increased with time from 3 to 15%, respectively. Our data indicated the zinc oxide nanospheres are safer for normal cells.

### Quantitative RT-PCR

**Figure 7:**
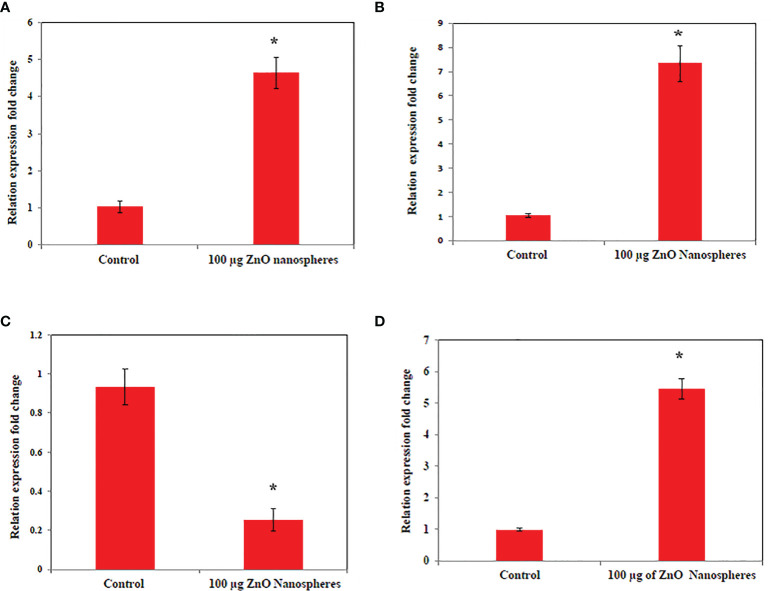
Quantitative real-time PCR measure mRNA levels of HuH7 exposed to 100 μg/ml of ZnO nanospheres (ZnO-NS) for 24 hour. *Statistically significant difference as compared with the controls (*P*, 0.05 for each). **(A)** P53 **(B)** Bax **(C)** Bcl2 **(D)** Cytochrome C.

The levels of apoptotic genes (p53, Bax, Bcl_2_, and cytochrome C) in the HuH7 cell were studied by treating the cell with ZnO nanospheres at a concentration 100 μg/ml for 24 h. The result revealed that ZnO nanospheres altered the expression of genes in HuH7 cells. The mRNA expression levels of tumor suppressor gene p53 (**Figure 7A**), pro-apoptotic gene Bax (**Figures 7B**, **D**) were significantly unregulated, while the expression of antiapoptotic gene BCL-2 (**Figure 7C**) was significantly down-regulated in ZnO NSs-treated cells as compared with the untreated control cells (P, 0.05 for each). Bax/Bcl_2_ ratio displayed the activity of ZnO- NSs show a good anticancer activity against HuH7 cell as **S1 Figure** displayed.

### Gene expression by flow cytometry

**Figure 8:**
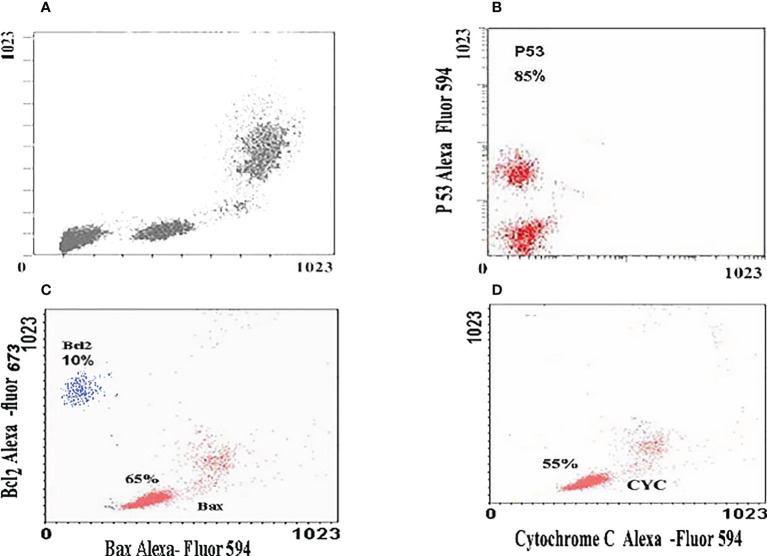
Immunoflorescent images of HuH7 exposed to 100 μg/ml of ZnO nanospheres (ZnO-NS) for 24 hour for up-regulation of P53, Bax, Bcl_2_ and Cytochrome C. **(A)** The immunoflorescent of huh7 remedied with DMSO **(B)** The immunoflorescent of P53 **(C)** The immunoflorescent of Bax and BcL_2_ **(D)** The immunoflorescent of Cytochrome C.

The relative expression of P53, Bax, and Bcl_2_ has been detected in all treated cells. Treatment of the HuH7 cells with ZnO nanospheres enhanced the relative expression of P53 significantly (P<0.05) by 85% (**Figure 8B**). Similarly, it increased the relative expression of Bax and Bcl_2_ to 65 and 10%, respectively (**Figure 8C**). Also, the relative expression of cytochrome C increased in the HuH7 cells treated with ZnO nanospheres by 55% (**Figure 8D**) compared to control cells treated with DMSO (**Figure 8A**). These findings indicated that ZnO nanospheres regulated cell cycles *via* stimulating the expression of P53 and Bax genes. Bcl2 was less detected than P53 and Bax.

### Western analysis

**Figure 9:**
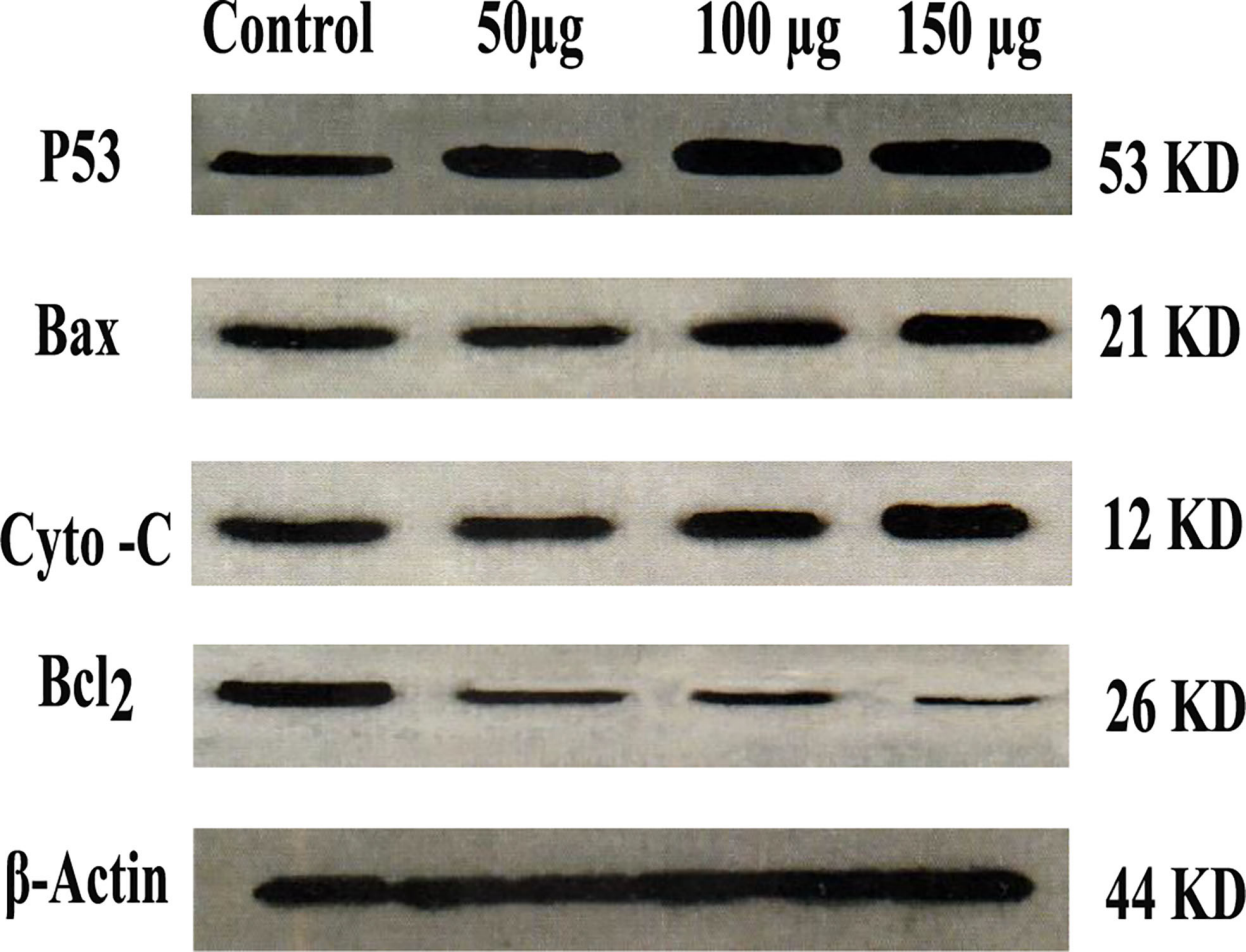
Western blot of P53, Bax, Cytochrome C and BcL_2_.

As shown in **Figure 9**, the western blot of the expressed protein demonstrated that P53, Bax, and cytochrome C were up-regulated as compared with control. While Bcl_2_ was down-regulated as compared with control, Zinc oxide nanosphere induces caspase-dependent apoptosis.

### Caspase-3 assay

**Figure 10:**
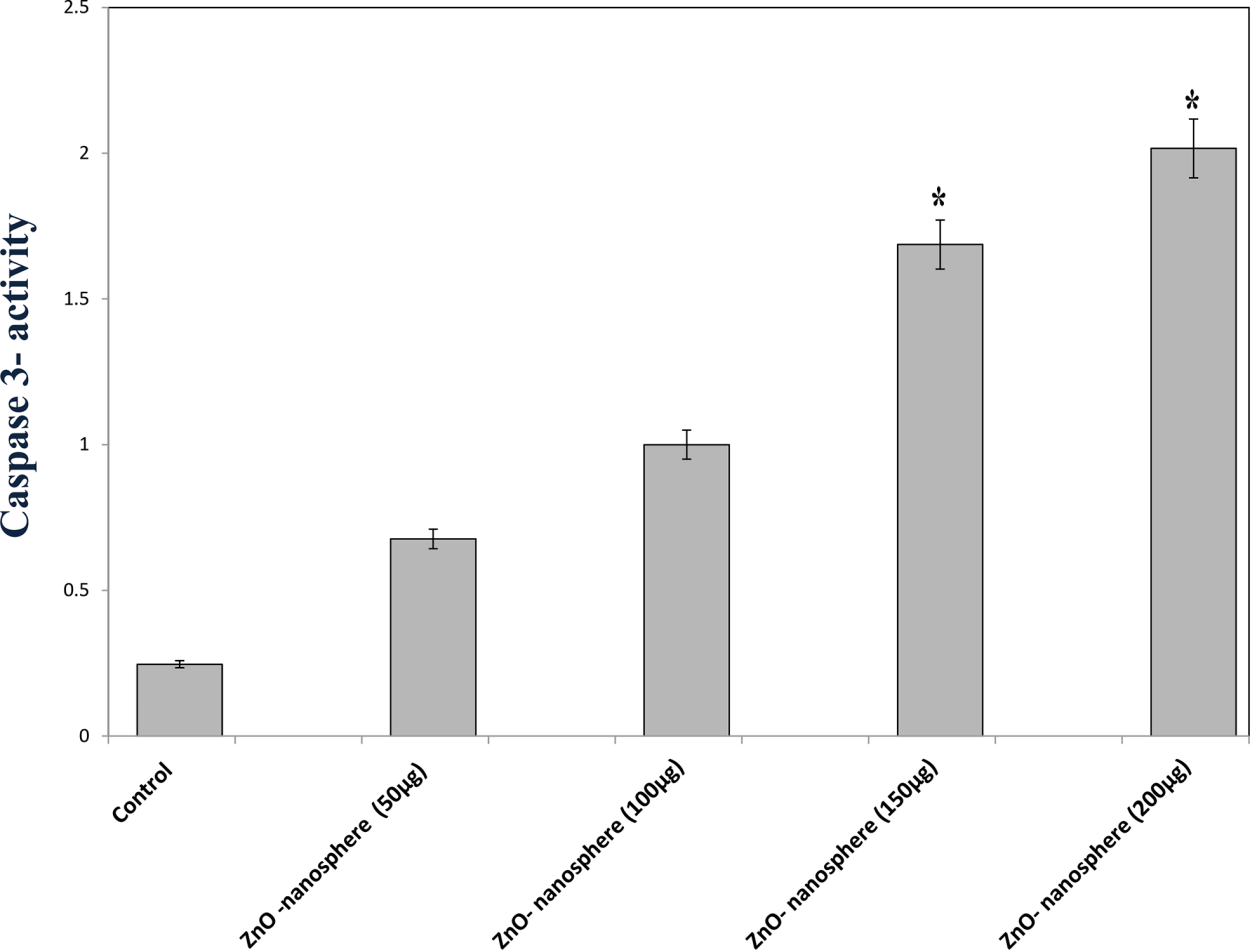
Caspase-3 measurement of ZnO nanospheres. *Statistically significant difference as compared with the controls (P> 0.05 for each).

Caspase-3 is one of the executioner caspase members. As **Figure 10** displayed, as the concentration of zinc oxide increased, the caspase 3 enzyme activity increased from 0.75 at a concentration of 50 µg to 2 at a concentration of 200 µg/ml. The data confirmed that zinc oxide nanospheres activated caspase-3 cleavage in a concentration-dependent manner.

### Oxidative stress and antioxidant biomarkers

**Figure 11:**
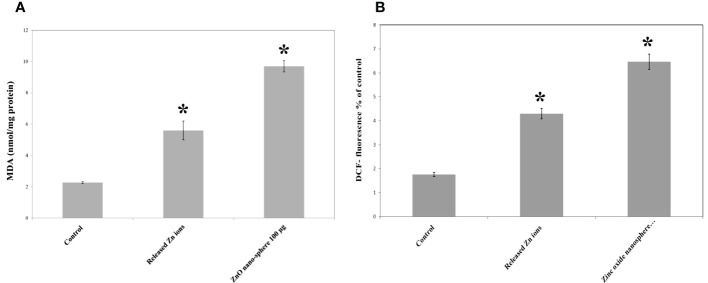
Oxidative stress and of human Hepatoma (HuH7) cells after treated with 100 μg/mL ZnO NPs and released Zn ion for 24 hour. *Statistically significant difference as compared with the controls (P *>* 0.05 for each). **(A)** ROS **(B)** MDA.

**Figure 12:**
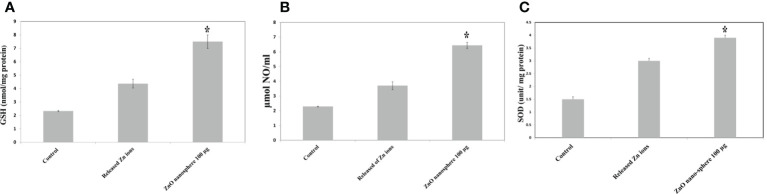
Antioxidant assay of human Hepatoma (HuH7) cells after treated with 100 μg/mL ZnO NSs and released Zn ion for 24 hour. *Statistically significant difference as compared with the controls (P *>* 0.05 for each). **(A)** Glutathione (GSH) **(B)** Superoxide dismutase (SOD) **(C)** Nitric oxide (NO).

The primary pathway required to trigger apoptosis mechanistically in a cancerous cell was to induce oxidant generation and antioxidant depletion. Therefore, the oxidants (ROS, LPO, and NO) and antioxidants (GSH and SOD) were studied in the HuH7 cells treated with 100 µg of ZnO nanospheres and Zn ^+2^ ions (released from 100 µg of ZnO nanospheres) after exposure to 24 hr. The results illustrated in **Figures 11A, B** showed that markers of oxidative stress (ROS and LPO) levels were significantly (P<0.05) higher in the HuH7 cells treated with ZnO nanospheres. As **Figures 12A-C** revealed, all the antioxidant indicators were increased due to the exposure to ZnO nanospheres and released Zn^+2^ ions. Nevertheless, the released Zn ^+2^ ions were less than ZnO nanospheres.

### Ultrastructure of HuH7 using a transmission electron microscope

**Figure 13:**
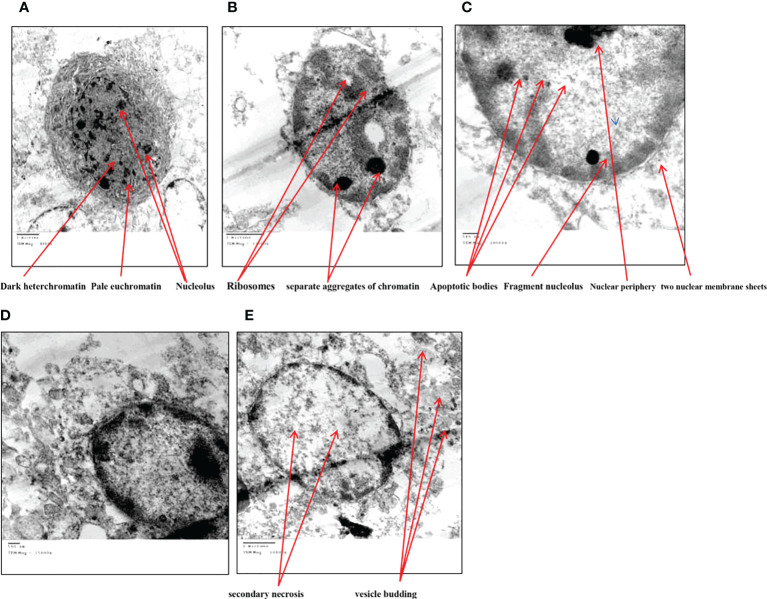
Transmission electron microscope of HuH7 exposed to 100 μg/ml of ZnO nanospheres (ZnO-NS) for 24 hour. **(A)** Control cell **(B)** Treated cell with to 100 μg/ml of ZnO nanospheres (ZnO-NS) for 3 hour. **(C)** Treated cell with to 100 μg/ml of ZnO nanospheres (ZnO-NS) for 6 hour. **(D)** Treated cell with to 100 μg/ml of ZnO nanospheres (ZnO-NS) for 12 hour. **(E)** Treated cell with to 100 μg/ml of ZnO nanospheres (ZnO-NS) for 24 hour.

TEM analysis of Huh7 cells remedied with zinc oxide nanospheres at different times demonstrated the typical features of apoptotic cell stages: To begin with, as shown in **Figure 13B**, the cells rounded up with the disappearance of cytoplasmic Secondly, as time increased, there were many changes in appearance because of chromatin condensation at the nuclear periphery, as **Figures 13C, D** revealed. Finally, as shown in **Figures 13E**, there is nuclear fragmentation (a vescicle budded from the nucleus) as compared with the control cell. As in **Figure 13A**. The control cell (untreated cell) displayed the morphology of regular cells with hyperchromatic nuclei and nuclear pleomorphism. As **Figure 13A** displayed, at high magnification, the cytoplasmic vacuoles were droplets. Similarly, other vacuoles with a double membrane existed, as **Figure 13E** showed. Consequently, chromatin condensation, as **Figure 13E** displays, Also, during the apoptosis process, mitochondria tend to be conserved till secondary necrosis is carried out during treatment of a Hepatoma cell model treated with zinc oxide nanosphere. The number of swollen mitochondria transfers to an apoptotic nuclear morphology within an intact plasma membrane.

## Discussion

Cancer therapy concerns the challenging aim of creating selective, effective, and safe therapy (44). MTX is the best antitumor chemotherapeutic drug (antineoplastic or cytotoxic) when used at high doses. However, those doses cause an adverse effect. Hence, scientists are working to find a novel drug with high performance and safety (45). Sorafenib is categorized as the most successful anticancer drug against hepatocellular carcinoma. Sorafenib, an oral multiple kinase inhibitor, significantly induces apoptosis in cancer model processes as well as inhibits tumor angiogenesis and cell proliferation to exert its anticancer activity, but it has severe cytotoxicity and may lead to adverse events (46). Previous research suggested that zinc oxide nanoparticles could be used as an anticancer drug agent by targeting cancerous cells and increasing cytotoxicity and cell death (47). Due to their less toxic nature toward normal cells, zinc oxide nanoparticles haven’t had any adverse effects on liver and renal tissues. Basically, the positive charge of a nanoparticle has more affinity to be engulfed by cells than neutral or negative nanoparticles. It is supposedly due to favorable electrostatic interactions with the negatively charged cell membrane (48). The surface of zinc oxide nanoparticles has a neutral hydroxyl group, which plays a central role in nanoparticle charging behavior (49). In an acidic medium, the proton more likely to bind to the surface of a nanoparticle may cause a positively charged surface (ZnOH^2+^). However, in an alkaline medium, protons moved away from the metal surface, inducing a negatively charged surface partly bonded oxygen atom (ZnO^−)^. Under physiological conditions (acidic medium of tumor cells), the isoelectric point value ranges from 9 to 10, indicating that zinc oxide nanoparticles have a strongly positive-charged surface (50). Cancer cell outer layer membranes are characterized by the presence of a large number of anionic phospholipids (48). As a result, ZnO-NPs may be attracted electrostatically to tumor tissues, increasing nanoparticle cellular uptake (51). In contrast, normal healthy cells are either charge-neutral or slightly positive, which shows insignificant binding to the NPs (52). Also, the circulation of nanoparticles with a size less than 100 nm takes a longer time with the ability to avoid clearance by the reticuloendothelial system, also increasing intratumor concentrations (53, 54).

### Mechanism of preparation of zinc oxide nanosphere

The preparation of zinc oxide nanospheres meets the concept of green chemistry. Green synthesis of nanomaterials is an interesting topic of material science research (55). This study was focused on the construction of zinc oxide nanospheres based on the accumulation of zinc ions on yeast extract, which was used as a bio-template (29). The plausible mechanism to explain the formation of zinc oxide nanospheres is that the dissolution of zinc acetate in distilled water released zinc ions that accumulated on the outer surface of yeast extract. Then, zinc ions formed on a spherical yeast extract. Afterwards, gradual thermal treatment leads to the formation of zinc oxide nanospheres and the removal of any yeast extract residuals. The physicochemical techniques such as FTIR, XR, DLS, and TEM emphasized the formation of ZnO nanoparticles in a spherical shape of 20 nm. ICP-AS is the routine method to evaluate the amount of zinc ions released from suspended ZnO nanospheres.

### Cytotoxicity and apoptosis of zinc oxide nanospheres against HuH7 and Vero cells

The antiproliferative effect of ZnO-NS and Zn^+2^ ions was determined using the MTT assay, which links directly to the mitochondrial enzymes (25). Our results showed a significant reduction in HuH7 viability in the treated groups compared with the nontreated group. Therefore, the ZnO nanospheres are more selective for cancerous cells than normal cells. The cell cycle is controlled by numerous mechanisms that ensure correct cell division. It is a transition between various phases mediated by different cellular proteins. It divides the cell into two consecutive processes, the majority of which are characterized by DNA replication and the segregation of replication chromosomes into two separate cells. Our findings show that ZnO nanospheres induced cell cycle arrest at the G2/M phase and aided in the inhibition of HuH7 cell proliferation. The cells can be able to initialize a cell cycle arrest in the presence or absence of P53. Interestingly, maintenance of CDK1 in its inhibitory phosphorylation state prevents cell cycle entry into mitosis. Also, P53 may play a central role in the regulation of the G2/M checkpoint and DNA damage-dependent increases in p53 (53). The Annexin V-FITC/PI double-staining assay indicates the occurrence of apoptosis. Treatment of HuH7 and Vero cells with different concentrations as well as different times confirmed that the ZnO nanospheres have a less adverse effect on normal cells. Importantly, the apoptosis process depends on the activation of caspase, which is considered a hallmark of apoptosis (56). Indeed, caspase results emphasized that caspase induces the apoptotic mechanism.

### Molecular mechanism of zinc oxide nanospheres against HuH7 and Vero cells

**Figure 14:**
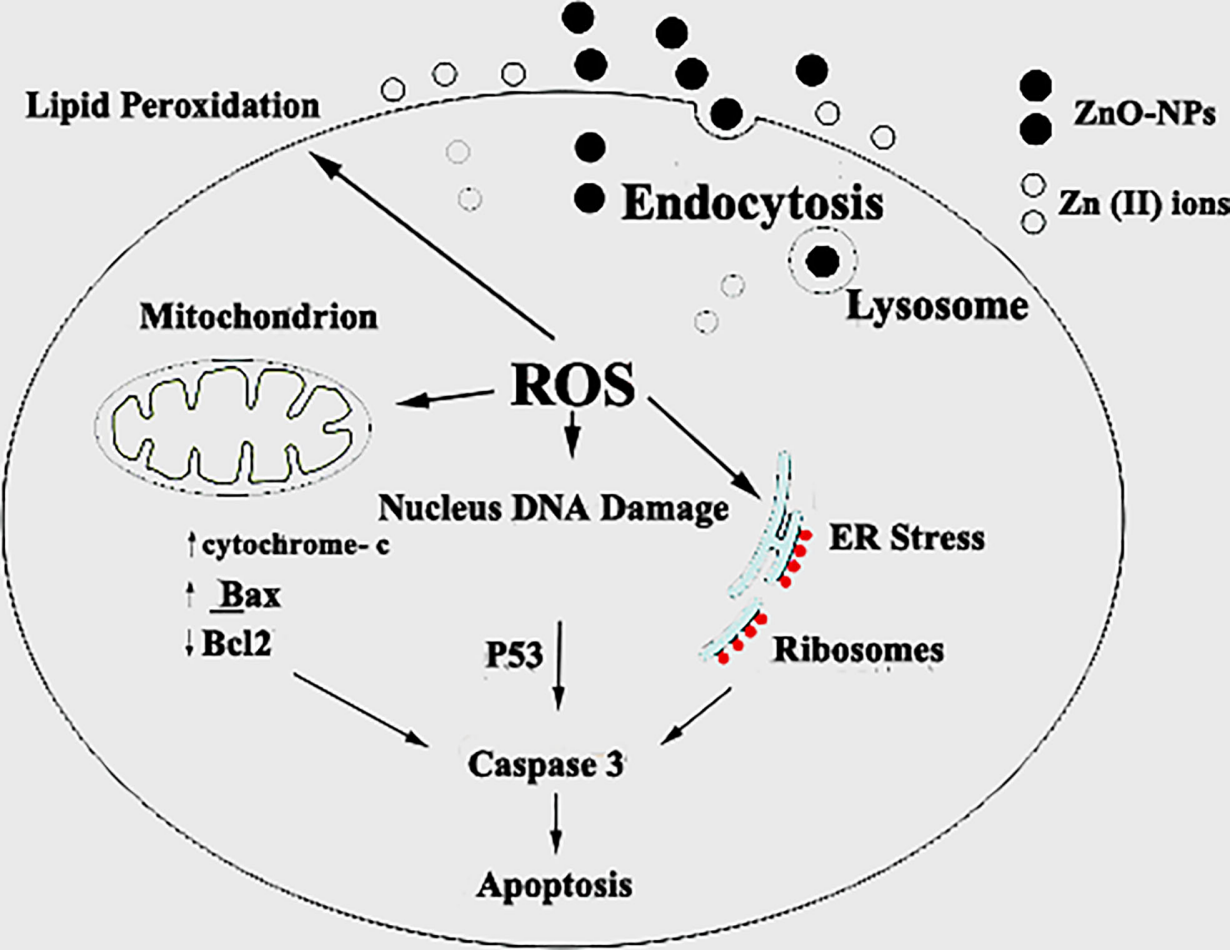
Schematic diagram of the probable mechanism of synergy of ZnO-NS. ZnO-NS are involved in reactive oxygen species (ROS) generation and increased cell wall lipid peroxidation (LPO) leading to cytotoxic and genotoxic effects.

Generally, zinc oxide nanoparticles may promote micropinocytosis. Hence, ROS toxicity after ZnO NPs uptake led to ZnO genotoxicity *in vitro* and *in vivo*. In particular, lacking normal TP53, which is the typical feature of small-cell lung cancer, should be taken into account for the use of ZnO treatment for small-cell lung cancer (27). Setyawati and colleagues reported that DLD1 and SW480 cells that have mutant TP53 produce more ROS by ZnO nanoparticles, and those cells were more susceptible to the cytotoxicity than NCM460 and HCT116 cells that have functional TP53 (57). Lee and colleagues reported that the ATM protein reacts to ROS and is necessary to phosphorylate TP53 (Ser 15) under ROS-induced oxidative stress. The loss of normal ATM protein results in deficiencies in mitochondrial function, autophagy, and intracellular protein aggregation (58). In addition, damaged mitochondria also produce intracellular ROS (27). RT-PCR, flowcytometry, and immunoblotting are common techniques to study the molecular mechanisms of apoptosis like P53, Bax, Bcl_2_, and cytochrome C. As **Figure 14** shows, there are a couple of mechanisms that meet the concept of apoptosis. The first one is the extrinsic pathway, which depends on ligation of the death receptor family, followed by the formation of the death-inducing signaling complex that leads to the activation of caspase (55). The second mechanism is the intrinsic pathway that depends on the pro and anti-apoptotic pathways of Bcl-2 family proteins. Furthermore, the mitochondrial cytochrome C release facilitates the formation of the apoptosome complex that cleaves and activates the effector caspase (56, 59). Also, there is another approach to inducing apoptosis by activating P53 to engage the mitochondria and release cytochrome C. Caspase 3 assay displayed that the mechanism of zinc oxide nanosphere is an extrinsic pathway that depends on ligation of the death receptor family, followed by the formation of the death-inducing signaling complex that leads to the activation of caspase (59).

### Oxidative stress of zinc oxide nanospheres

Oxidative stress, including H_2_O_2_, ***^·^*** OH, and. O^2 −,^ may react with nucleophilic centers, leading to DNA fragmentation and apoptosis upregulation, ultimately leading to carcinoma cell death (25). Our findings agree with Bai et al. (60) who reported a significant increase in the DNA damage in the ZnO-NP-treated group compared with the nontreated SEC group. Treatment of HuH7 cells with ZnO nanospheres caused an increase in pro-oxidants (ROS and LPO). According to Finucane et al. (1999), ZnO nanoparticles generate reactive oxygen species that activate the peroxidation reactions (55). Also, as **Figure 14** shows, the elevated ROS level can inflict direct damage to lipids (61, 62). Because of its reaction with thiobarbituric acid (TBA), MDA is regarded as a useful biomarker for the peroxidation of omega-3 and omega-6 fatty acids. Similarly, excessive Zn^2+^ ions trigger harmful oxidative stress. According to Lee et al. (2018), zinc deficiency or excess can cause cellular oxidative stress (63, 64). Hence, liberated zinc ions released from suspended zinc oxide nanospheres medium indirectly contributed to stimulating the oxidative stress on HuH7 cells. Moreover, GSH, SOD, and NO attenuated the deleterious effect of ROS. GSH was an important ROS scavenger. An enzyme catalyses the transformation of the highly reactive O^-2^ to H_2_O_2_. Also, NO is a special scavenger against ROS (61). Our findings support the findings of Xia et al. (65), who found that ZnO-NPs increase cancer cytotoxicity and cell death. Other studies have shown that increased ROS levels cause oxidative damage to cellular structures and decrease antioxidant enzymes SOD and CAT activity in tumor tissue compared to the SEC group, as SOD catalyzes the dismutation of superoxide anion (O_2_) to H_2_O_2_ and O_2_, and the CAT enzyme reduces H_2_O_2_ to H_2_O. Interestingly, treatment with ZnO-NPs revealed a marked increase in MDA levels, which indicates lipid peroxidation and depletion of GSH in ESC tissues (25). Also, some studies have reported that both solid ZnO-NPs as well as dissolved Zn ions are responsible for the toxic responses of exposed cells (62). Heim et al. (66) found higher values of Zn^2+^ in the nucleus compared to the cytoplasm, indicating the enrichment of dissolved Zn^2+^. However, contrary to this observation, Sharma et al. (67) also addressed the Zn^2+^ contribution towards the ZnO-NPs toxicity These authors observed that toxic responses from exposure to ZnO-NPs are due to the NPs per se rather than the Zn^2+^ released from them.

### Ultrastructural analysis of zinc oxide nanospheres against hepatoma

The transmission electron microscope (TEM) has become a standard method for describing and analyzing the ultrastructure of inner cells and organelles in physiological and pathological conditions (68). TEM is the most powerful morphological method to describe apoptosis and necrosis. Its images are qualitative and static. As a result, the best method for studying apoptotic and necrotic phenomena should be carried out at different times, beginning with their early-stage appearance (69). Apoptosis is a regulated process that controls many morphological and physiological pathways in an active manner. It’s characterized by various biochemical processes with cascades that respond to the change in the morphology of the cell that may lead to the death of the cell. Multi-factors control the type of cell termination. The main role of apoptosis is caspase activation, which influences the level of intracellular ATP. As a result, mitochondria are concerned with this type of cell death. During the apoptosis process, the cell size shrinks and loses connectivity with adjacent cells. The cells miss their surface elements such as microvilli and cell-to-cell linkage. Because of the liberation of the cell fluid, the cytoplasm will condensate, leading to a change in the cell volume. Therefore, the convolution of the nuclear and cellular outlines Initially, condensed chromatin tends toward the formation of cup-shaped masses beneath the nuclear envelope to fill thymocyte cells that occupy most of the nuclear volume (56). Finally, the apoptosis process will undergo progressive fragmentation, leading to the formation of several plasma membrane-bound apoptotic bodies, including nuclear and/or cytoplasmic elements (70). On the other hand, necrosis is another form of cell deletion. In the necrosis process, the plasma membrane will be permeable rapidly. Generally, cell hydration and swelling, as well as organelle disruption, appear. Inflammation is caused by the release of cytosolic components into the extracellular space (71–74). In contrast to the apoptosis process, the nucleus shape appears well in the early stages. Transmission electron microscopy analysis of HuH7 cells exposed to zinc oxide nanospheres demonstrated the stages of apoptosis. However, vacuolization was also demonstrated. Some of these vacuoles were double membrane bound vesicles, as shown in **Figure 14**. These vacuoles include residual cristae structure and may be due to mitochondrial swelling. As Harris and Ralph (1985) (75) reported, the apoptotic condition can induce a cell to differentiate. That can transfer the cell to a partial differentiation state (75–80).

## Conclusion

We conclude empirical evidence of the genotoxic anticancer effects of ZnO in hepatic cancer cells, which may be executed safer and more effective antitumor activity, leading to SEC growth reduction as shown in **Figure 14**, with a low cytotoxic effect on normal cells. Therefore, new therapeutic strategies for cancer treatment, including ZnO-NS, could be developed. Moreover, long-term toxicity studies are required to rule out any long-term side effects. Overall, the data showed that ZnO nanospheres might have a good choice as a potential candidate as an anti-tumor agent.

## Data availability statement

The original contributions presented in the study are included in the article/**Supplementary Material**. Further inquiries can be directed to the corresponding author.

## Author contributions

Conceptualization: AH and AM; formal analysis: AH; funding acquisition: AH, FA-S, ED, FA, and HAA; investigation: AH, AM, and AE; investigation: MH, Wagih M. Adelhay, TM, FAE-F, and EF; methodology: AH, TA, and EM; project administration: AH and AE; Resource: AE; supervision: AH; Validation: AH, AE, and AM; visualization: AE, ES, MZ, HEA, MA, and AH; writing original draft: AH and AM; writing -review edition; AH and AM. All author approved the final version of the manuscript.

## Funding

This work was fully supported by Taif University Researchers supporting project number (TURSP-2020/113), Taif University, Saudi Arabia.

## Acknowledgments

The authors would like to thank the Deanship of Scientific Research at Taif University for funding this work through Taif University Researchers Supporting Project number (TURSP - 2020/113), Taif University, Taif, Saudi Arabia.

## Conflict of interest

The authors declare that the research was conducted in the absence of any commercial or financial relationships that could be construed as a potential conflict of interest.

## Publisher’s note

All claims expressed in this article are solely those of the authors and do not necessarily represent those of their affiliated organizations, or those of the publisher, the editors and the reviewers. Any product that may be evaluated in this article, or claim that may be made by its manufacturer, is not guaranteed or endorsed by the publisher.

## Supplementary material

Supplemental image S1 Bax/bcl2 ratio.

## References

[1] Bruix J Raoul JL Sherman M Mazzaferro V Bolondi L Craxi A. Efficacy and safety of sorafenib in patients with advanced hepatocellular carcinoma: subanalyses of a phase III trial. J Hepatol (2012) 57(4):821–9. doi: 10.1016/j.jhep.2012.06.014 PMC12261288 22727733

[2] Jemal A Bray F Center MM Ferlay J Ward E Forman D. Global cancer statistics. CA: Cancer J Clin (2011) 61(2):69–90. doi: 10.3322/caac.20107 21296855

[3] Sawada Y Yoshikawa T Nobuoka D Shirakawa H Kuronuma T Motomura Y. Phase I trial of a glypican-3–derived peptide vaccine for advanced hepatocellular carcinoma: immunologic evidence and potential for improving overall survival. Clin Cancer Res (2012) 18(13):3686–3696. doi: 10.1158/1078-0432.CCR-11-3044 22577059

[4] Feo F Simile MM Pascale RM. Focal loss of long non-coding RNA-PRAL, as determinant of cell function and phenotype of hepatocellular carcinoma. Ann Trans Med (2016) 4(9):183. doi: 10.21037/atm.2016.03.47 PMC4876269 27275496

[5] Ries LA Harkins D Krapcho M Mariotto A Miller BA Feuer EJ. SEER cancer statistics review, 1975–2005. Bethesda, MD: National Cancer Institute (2008), 2999. http://seer.cancer.gov/csr/1975_2005.

[6] Nakabayashi H Taketa K Miyano K Yamane T Sato J. Growth of human hepatoma cell lines with differentiated functions in chemically defined medium. Cancer Res (1982) 42(9):3858–63. 6286115

[7] Goldring CE Kitteringham NR Jenkins R Lovatt CA Randle LE Abdullah A. Development of a transactivator in hepatoma cells that allows expression of phase I, phase II, and chemical defense genes. Am J Physiology-Cell Physiol (2006) 290(1):C104–15. doi: 10.1152/ajpcell.00133.2005 16135546

[8] Yoon HI Seong J. Multimodality treatment involving radiotherapy for advanced liver-confined hepatocellular carcinoma. Oncology (2014) 87(Suppl.1):90–8. doi: 10.1159/000368151 25427739

[9] Yeo W Mok TS Zee B Leung TW Lai PB Lau WY. A randomized phase III study of doxorubicin versus cisplatin/interferon α-2b/doxorubicin/fluorouracil (PIAF) combination chemotherapy for unresectable hepatocellular carcinoma. J Natl Cancer Institute (2005) 97(20):1532–8. doi: 10.1093/jnci/dji315 16234567

[10] Gish RG Porta C Lazar L Ruff P Feld R Croitoru A. Phase III randomized controlled trial comparing the survival of patients with unresectable hepatocellular carcinoma treated with nolatrexed or doxorubicin. J Clin Oncol (2007) 25(21):3069–75. doi: 10.1200/JCO.2006.08.4046 17634485

[11] Wang XQ Ongkeko WM Chen L Yang ZF Lu P Chen KK. Octamer 4 (Oct4) mediates chemotherapeutic drug resistance in liver cancer cells through a potential Oct4–AKT–ATP-binding cassette G2 pathway. Hepatology (2010) 52(2):528–39. doi: 10.1002/hep.23692 20683952

[12] Ohta K Hoshino H Hata K Wang J Huang S Menon V. MicroRNA mir-93 activates oncogenic c-Met/PI3K/Akt pathway targeting PTEN in hepatocellular carcinoma. Cancer Res (2014) 74(19_Supplement):4686–86. doi: 10.1158/1538-7445.AM2014-4686

[13] Siegel R Ward E Brawley O Jemal A. The impact of eliminating socioeconomic and racial disparities on premature cancer deaths. Ca-a Cancer J Clin (2011) 61(4):212–36. doi: 10.3322/caac.20121 21685461

[14] Iwamoto H Torimura T Nakamura T Hashimoto O Inoue K Kurogi J. Metronomic s-1 chemotherapy and vandetanib: an efficacious and nontoxic treatment for hepatocellular carcinoma. Neoplasia (2011) 13(3):187–97. doi: 10.1593/neo.101186 PMC3050862 21390182

[15] Peck-Radosavljevic M. Drug therapy for advanced-stage liver cancer. Liver Cancer (2014) 3(2):125–31. doi: 10.1159/000343868 PMC4057791 24945003

[16] Schwarz M Andrade-Navarro MA Gross A. Mitochondrial carriers and pores: key regulators of the mitochondrial apoptotic program? Apoptosis (2007) 12(5):869–76. doi: 10.1007/s10495-007-0748-2 17453157

[17] Hassan A Elebeedy D Matar ER Fahmy Mohamed Elsayed A Abd El Maksoud AI. Investigation of angiogenesis and wound healing potential mechanisms of zinc oxide nanorods. Front Pharmacol (2021) 12:661217. doi: 10.3389/fphar.2021.661217 34721007 PMC8552110

[18] An WG Kanekal M Simon MC Maltepe E Blagosklonny MV Neckers LM. Stabilization of wild-type p53 by hypoxia-inducible factor 1α. Nature (1998) 392(6674):405–8. doi: 10.1038/32925 9537326

[19] Fakruddin M Hossain Z Afroz H. Prospects and applications of nanobiotechnology: a medical perspective. J nanobiotechnology (2012) 10(1):1–8. doi: 10.1186/1477-3155-10-31 22817658 PMC3422163

[20] Siddiqui SA Or Rashid M Uddin M Robel FN Hossain MS Haque M. Biological efficacy of zinc oxide nanoparticles against diabetes: a preliminary study conducted in mice. Bioscience Rep (2020) 40(4):BSR20193972. doi: 10.1042/BSR20193972 PMC7138905 32207527

[21] Sharaf EM Hassan A AL-Salmi FA Albalwe FM Albalaw HMR Darwish DB. Synergistic antibacterial activity of compact silver/magnetite core-shell nanoparticles core shell against gram-negative foodborne pathogens. Front Microbiol (2022) 13:929491. doi: 10.3389/fmicb.2022.929491 36118244 PMC9478199

[22] Nagajyothi PC Cha SJ Yang IJ Sreekanth TVM Kim KJ Shin HM. Antioxidant and anti-inflammatory activities of zinc oxide nanoparticles synthesized using polygala tenuifolia root extract. J Photochem Photobiol B: Biol (2015) 146:10–7. doi: 10.1016/j.jphotobiol.2015.02.008 25777265

[23] Hassan A Sorour NM El-Baz A Shetaia Y. Simple synthesis of bacterial cellulose/ magnetite nanoparticles composite for the removal of antimony from aqueous solution. Internati onal J Environ Sci Technol (2019) 16:1433–48. doi: 10.1007/s13762-018-1737-4

[24] Anjum S Hashim M Malik SA Khan M Lorenzo JM Abbasi BH. Recent advances in zinc oxide nanoparticles (ZnO NPs) for cancer diagnosis, target drug delivery, and treatment. Cancers (2021) 13:4570. doi: 10.3390/cancers13184570 34572797 PMC8468934

[25] Nabil A Elshemy MM Asem M Abdel-Motaal M Gomaa HF Zahran F. Zinc oxide nanoparticle synergizes sorafenib anticancer efficacy with minimizing its cytotoxicity. oxidative medicine and cellular longevity, 2020.S.-e. jin and h. e. jin, “Synthesis, characterization, and threedimensional structure generation of zinc oxide-based nanomedicine for biomedical applications,”. Pharmaceutics (2020) 11(11):575. doi: 10.1155/2020/1362104 PMC7275957 32566073

[26] Kielbik J Kaszewski Dominiak B Damentko M Serafińska I Rosowska J. Preliminary studies on biodegradable zinc oxide nanoparticles doped with fe as a potential form of iron delivery to the living organism. Nanoscale Res Lett (2019) 14(1):373. doi: 10.1186/s11671-019-3217-2 31823131 PMC6904721

[27] Tanino R Amano Y Tong X Sun R Tsubata Y Harada M. Anticancer activity of ZnO nanoparticles against human small-cell lung cancer in an orthotopic mouse ModelZnO nanoparticles inhibit growth of small-cell lung cancer. Mol Cancer Ther (2020) 19(2):502–12. doi: 10.1158/1535-7163.MCT-19-0018 31784453

[28] Fujihara J Tongu M Hashimoto H Yamada T Kimura-kataoka K Yasuda T. Distribution and toxicity evaluation of ZnO dispersion nanoparticles in single intravenously exposed mice. J Med Invest (2015) 62:45–50. doi: 10.2152/jmi.62.45 25817283

[29] Wang J Deng X Zhang F Chen D Ding W. ZnO nanoparticle-induced oxidative stress triggers apoptosis by activating JNK signaling pathway in cultured primary astrocytes. Nanoscale Res Lett (2014) 9(1):1–12. doi: 10.1186/1556-276X-9-117 24624962 PMC3995614

[30] Bao SJ Lei C Xu MW Cai CJ Jia DZ. Environment-friendly biomimetic synthesis of TiO2 nanomaterials for photocatalytic application. Nanotechnology (2012) 23(20):205601. doi: 10.1088/0957-4484/23/20/205601 22543361

[31] Tada-Oikawa S Ichihara G Suzuki Y Izuoka K Wu W Yamada Y. Zn (II) released from zinc oxide nano/micro particles suppresses vasculogenesis in human endothelial colony-forming cells. Toxicol Rep (2015) 2:692–701. doi: 10.1016/j.toxrep.2015.04.003 28962405 PMC5598154

[32] Mosmann T. Rapid colorimetric assay for cellular growth and survival: application to proliferation and cytotoxicity assays. J Immunol Methods (1983) 65(1-2):55–63. doi: 10.1016/0022-1759(83)90303-4 6606682

[33] Pal A Alam S Mittal S Arjaria N Shankar J Kumar M. UVB irradiation-enhanced zinc oxide nanoparticles-induced DNA damage and cell death in mouse skin. Mutat Research/Genetic Toxicol Environ Mutagenesis (2016) 807:15–24. doi: 10.1016/j.mrgentox.2016.06.005 27542711

[34] Hamouda RA Abd El Maksoud AI Wageed M Alotaibi AS Elebeedy D Khalil H. Characterization and anticancer activity of biosynthesized Au/Cellulose nanocomposite from chlorella vulgaris. Polymers (2021) 13:3340. doi: 10.3390/polym13193340 34641156 PMC8512388

[35] Khella KF Abd El Maksoud AI Hassan A Abdel-Ghany SE Elsanhoty RM Aladhadh MA. Carnosic acid encapsulated in albumin nanoparticles induces apoptosis in breast and colorectal cancer cells. Molecules (2022) 27(13):4102. doi: 10.3390/molecules27134102 35807348 PMC9268188

[36] Taher RF Al-Karmalawy AA Abd El Maksoud AI Khalil H Hassan A El-Khrisy EDA. Two new flavonoids and anticancer activity of hymenosporum flavum: *in vitro* and molecular docking studies. J Herbmed Pharmacol (2021) 10(4):443–58. doi: 10.34172/jhp.2021.52

[37] Akhtar MJ Ahamed M Kumar S Khan MM Ahmad J Alrokayan SA. Zinc oxide nanoparticles selectively induce apoptosis in human cancer cells through reactive oxygen species. Int J nanomedicine (2012) 7:845. doi: 10.2147/IJN.S29129 22393286 PMC3289443

[38] Del Principe MI Dal Bo M Bittolo T Buccisano F Rossi FM Zucchetto A. Clinical significance of bax/bcl-2 ratio in chronic lymphocytic leukemia. haematologica (2016) 101(1):77. doi: 10.3324/haematol.2015.131854 26565002 PMC4697894

[39] Ohkawa H Ohishi N Yagi K. Assay for lipid peroxides in animal tissues by thiobarbituric acid reaction. Analytical Biochem (1979) 95(2):351–8. doi: 10.1016/0003-2697(79)90738-3 36810

[40] Mbazima VG Mokgotho MP February F Rees DJG Mampuru LJM. Alteration of bax-to-Bcl-2 ratio modulates the anticancer activity of methanolic extract of commelina benghalensis (Commelinaceae) in jurkat T cells. Afr J Biotechnol (2008) 7(20).

[41] Ellman GL. Tissue sulfhydryl groups. Arch Biochem biophysics (1959) 82(1):70–7. doi: 10.1016/0003-9861(59)90090-6 13650640

[42] Green LC Wagner DA Glogowski J Skipper PL Wishnok JS Tannenbaum SR. Analysis of nitrate, nitrite, and [15N] nitrate in biological fluids. Analytical Biochem (1982) 126(1):131–8. doi: 10.1016/0003-2697(82)90118-X 7181105

[43] Bozzola JJ Russell LD. Electron microscopy: principles and techniques for biologists. Jones & Bartlett Learning (1999).

[44] Astolfi L Ghiselli S Guaran V Chicca M Simoni E Olivetto E. Correlation of adverse effects of cisplatin administration in patients affected by solid tumours: a retrospective evaluation. Oncol Rep (2013) 29(4):1285–92. doi: 10.3892/or.2013.2279 PMC3621656 23404427

[45] Nabil A Elshemy MM Asem M Abdel-Motaal M Gomaa HF Zahran F. Zinc oxide nanoparticle synergizes sorafenib anticancer efficacy with minimizing its cytotoxicity. Oxid Med Cell Longevity (2020) 2020:11. doi: 10.1155/2020/1362104 PMC7275957 32566073

[46] Ekenel M Iwamoto FM Ben-Porat LS Panageas KS Yahalom J DeAngelis LM. Primary central nervous system lymphoma: the role of consolidation treatment after a complete response to high-dose methotrexate based chemotherapy. Cancer Res (2008) 113(5):1025–31. doi: 10.1002/cncr.23670 18618509

[47] Liu C-Y Tseng LM Su JC Chang KC Tai WT Shiau CW. Novel sorafenib analogues induce apoptosis through SHP-1 dependent STAT3 inactivation in human breast cancer cells. Breast Cancer Res (2013) 15(4):3254. doi: 10.1186/bcr3457 PMC3978748 23938089

[48] Rasmussen JW Martinez E Louka P Wingett DG. Zinc oxide nanoparticles for s elective destruction of tumor cells and potential for drug delivery applications. Expert Opin Drug Delivery (2010) 7(9):1063–77. doi: 10.1517/17425247.2010.502560 PMC2924765 20716019

[49] Kundu M Sadhukhan P Ghosh N Chatterjee S Manna P Das J. pH-responsive and targeted delivery of curcumin via phenylboronic acid- functionalized ZnO nanoparticles for breast cancer therapy. J Advanced Res (2019) 18:161–72. doi: 10.1016/j.jare.2019.02.036 PMC6479012 31032117

[50] Forest V Pourchez J. Preferential binding of positive nanoparticles on cell membranes is due to electrostatic interactions: a too simplistic explanation that does not take into account the nanoparticle protein corona. Materials Sci Eng (2017) 70:889–96. doi: 10.1016/j.msec.2016.09.016 27770966

[51] Qu F Morais PC. The pH dependence of the surface charge density in oxide-based semiconductor nanoparticles immersed in aqueous solution. IEEE Trans Magnetics (2001) 37:2654–6. doi: 10.1109/20.951264

[52] Degen A Kosec M. Effect of pH and impurities on the surface charge of zinc oxide in aqueous solution. J Eur Ceramic Soc (2000) 20(6):667–73. doi: 10.1016/S0955-2219(99)00203-4

[53] Peetla C Vijayaraghavalu S Labhasetwar V. Biophysics of cell membrane lipids in cancer drug resistance: implications for drug transport and drug delivery with nanoparticles. Advanced Drug Delivery Rev (2013) 65(13-14):1686–1698. doi: 10.1016/j.addr.2013.09.004 PMC3840112 24055719

[54] Le W Chen B Cui Z Liu Z Shi D. Detection of cancer cells based on glycolytic-regulated surface electrical charges. Biophysics Rep (2019) 5(1):10–8. doi: 10.1007/s41048-018-0080-0

[55] Brannon-Peppas L Blanchette JO. Nanoparticle and targeted systems for cancer therapy. Advanced Drug Delivery Rev (2004) 56(11):1649–59. doi: 10.1016/j.addr.2004.02.014 15350294

[56] Qu Y Kang M Cheng X Zhao J. Chitosan-coated titanium dioxide-embedded paclitaxel nanoparticles enhance anti-tumor efficacy against osteosarcoma. Front Oncol (2020) 10:1837. doi: 10.3389/fonc.2020.577280 PMC7509149 33014883

[57] Setyawati MI Tay CY Leong DT. Effect of zinc oxide nanomaterials-induced oxidative stress on the p53 pathway. Biomaterials (2013) 34:10133–42.41. doi: 10.1016/j.biomaterials.2013.09.024 24090840

[58] Lee JH Mand MR Kao CH Zhou Y Ryu SW Richards AL. ATM Directs DNA damage responses and proteostasis via genetically separable pathways. Sci Signal (2018) 11:eaan5598. doi: 10.1126/scisignal.aan5598 29317520 PMC5898228

[59] Vermeulen K Van Bockstaele DR Berneman ZN. The cell cycle: a review of regulation, deregulation and therapeutic targets in cancer. Cell proliferation (2003) 36(3):131–49. doi: 10.1046/j.1365-2184.2003.00266.x PMC6496723 12814430

[60] Bai D-P Zhang XF Zhang GL Huang YF Gurunathan S. Zinc oxide nanoparticles induce apoptosis and autophagy in human ovarian cancer cells. Int J Nanomedicine (2017) 12:6521–35. doi: 10.2147/IJN.S140071 PMC5592910 28919752

[61] Zhuo Z Hu J Yang X Chen M Lei X Deng L. Ailanthone inhibits Huh7 cancer cell growth *via* cell cycle arrest and apoptosis *in vitro* and *in vivo* . Sci Rep (2015) 5(1):1–15. doi: 10.1038/srep16185 PMC4630794 26525771

[62] Luo X Budihardjo I Zou H Slaughter C Wang X. Bid, a Bcl2 interacting protein, mediates cytochrome c release from mitochondria in response to activation of cell surface death receptors. Cell (1998) 94(4):481–90. doi: 10.1016/S0092-8674(00)81589-5 9727491

[63] Li H Zhu H Xu CJ Yuan J. Cleavage of BID by caspase 8 mediates the mitochondrial damage in the fas pathway of apoptosis. Cell (1998) 94(4):491–501. doi: 10.1016/S0092-8674(00)81590-1 9727492

[64] Julia H Eva F Nawaz MT Anke K Ulf RH Christoph B. Genotoxic effects of zinc oxide nanoparticles. Nanoscale (2015) 7:8931–8. doi: 10.1039/C5NR01167A 25916659

[65] Xia T Kovochich M Brant J Hotze M Sempf J Oberley T. Comparison of the abilities of ambient and manufactured nanoparticles to induce cellular toxicity according to an oxidative stress paradigm. Nano Lett (2006) 6(8):1794–807. doi: 10.1021/nl061025k 16895376

[66] Song W Zhang J Guo J Ding F Li L Sun Z. Role of the dissolved zinc ion and reactive oxygen species in cytotoxicity of ZnO nanoparticles. Toxicol Lett (2009) 199(3):389–97. doi: 10.1016/j.toxlet.2010.10.003 20934491

[67] Sharma V Anderson D Dhawan A. Zinc oxide nanoparticles induce oxidative DNA damage and ROS-triggered mitochondria mediated apoptosis in human liver cells (HepG2). Apoptosis (2012) 17:852–70. doi: 10.1007/s10495-012-0705-6 22395444

[68] Gross A Yin XM Wang K Wei MC Jockel J Milliman C. Caspase cleaved BID targets mitochondria and is required for cytochrome c release, while BCL-XL prevents this release but not tumor necrosis factor-R1/Fas death. J Biol Chem (1999) 274(2):1156–63. doi: 10.1074/jbc.274.2.1156 9873064

[69] Finucane DM Bossy-Wetzel E Waterhouse NJ Cotter TG Green DR. Bax-induced caspase activation and apoptosis *via* cytochromec release from mitochondria is inhibitable by bcl-xL. J Biol Chem (1999) 274(4):2225–33. doi: 10.1074/jbc.274.4.2225 9890985

[70] Moldovan L Moldovan NI. Oxygen free radicals and redox biology of organelles. Histochem Cell Biol (2004) 122(4):395–412. doi: 10.1007/s00418-004-0676-y 15452718

[71] Schneider C Boeglin WE Yin H Porter NA Brash AR. Intermolecular peroxyl radical reactions during autoxidation of hydroxy and hydroperoxy arachidonic acids generate a novel series of epoxidized products. Chem Res Toxicol (2008) 21(4):895–903. doi: 10.1021/tx700357u 18324788

[72] Lee SR. Critical role of zinc as either an antioxidant or a prooxidant in cellular systems. Oxid Med Cell Longevity (2018) 2018:11. doi: 10.1155/2018/9156285 PMC5884210 29743987

[73] Bai W Zhang Z Tian W He X Ma Y Zhao Y. Toxicity of zinc oxide nanoparticles to zebrafish embryo: a physicochemical study of toxicity mechanism. J Nanoparticle Res (2010) 12(5):1645–54. doi: 10.1007/s11051-009-9740-9

[74] Burattini S Ferri P Battistelli M Curci R Luchetti F Falcieri E. C2C12 murine myoblasts as a model of skeletal muscle development: morpho-functional characterization. Eur J Histochem (2004) 48(3), 223–34. doi: 10.4081/891 15596414

[75] Harris P Ralph P. Human leukemic models of myelomonocytic development: a review of the HL-60 and U937 cell lines. J leukocyte Biol (1985) 37(4):407–22. doi: 10.1002/jlb.37.4.407 3855947

[76] Di Baldassarre A Secchiero P Grilli A Celeghini C Falcieri E Zauli G. Morphological features of apoptosis in hematopoietic cells belonging to the T-lymphoid and myeloid lineages. Cell Mol Biol (2000) 46(1):153–61. 10726981

[77] Falcieri E Luchetti F Burattini S Canonico B Santi S Papa S. Lineage-related sensitivity to apoptosis in human tumor cells undergoing hyperthermia. Histochem Cell Biol (2000) 113(2):135–44. doi: 10.1007/s004180050016 10766266

[78] Golstein P Kroemer G. Cell death by necrosis: towards a molecular definition. Trends Biochem Sci (2007) 32(1):37–43. doi: 10.1016/j.tibs.2006.11.001 17141506

[79] Klöditz K Fadeel B. Three cell deaths and a funeral: macrophage clearance of cells undergoing distinct modes of cell death. Cell Death Discovery (2019) 5(1):65. doi: 10.1038/s41420-019-0146-x 30774993 PMC6368547

[80] Llovet JM Hernandez-Gea V. Hepatocellular carcinoma: reasons for phase III failure and novel perspectives on trial design. Clin Cancer Res (2014) 20(8):2072–9. doi: 10.1158/1078-0432.CCR-13-0547 24589894

