# Supplementary figures and images for: Ultrastructural analysis of zinc oxide nanospheres enhances anti-tumor efficacy against Hepatoma
Source: Front Oncol. 2022 Oct 27;12:933750. doi: 10.3389/fonc.2022.933750 (PMC9706544; doi:10.3389/fonc.2022.933750)

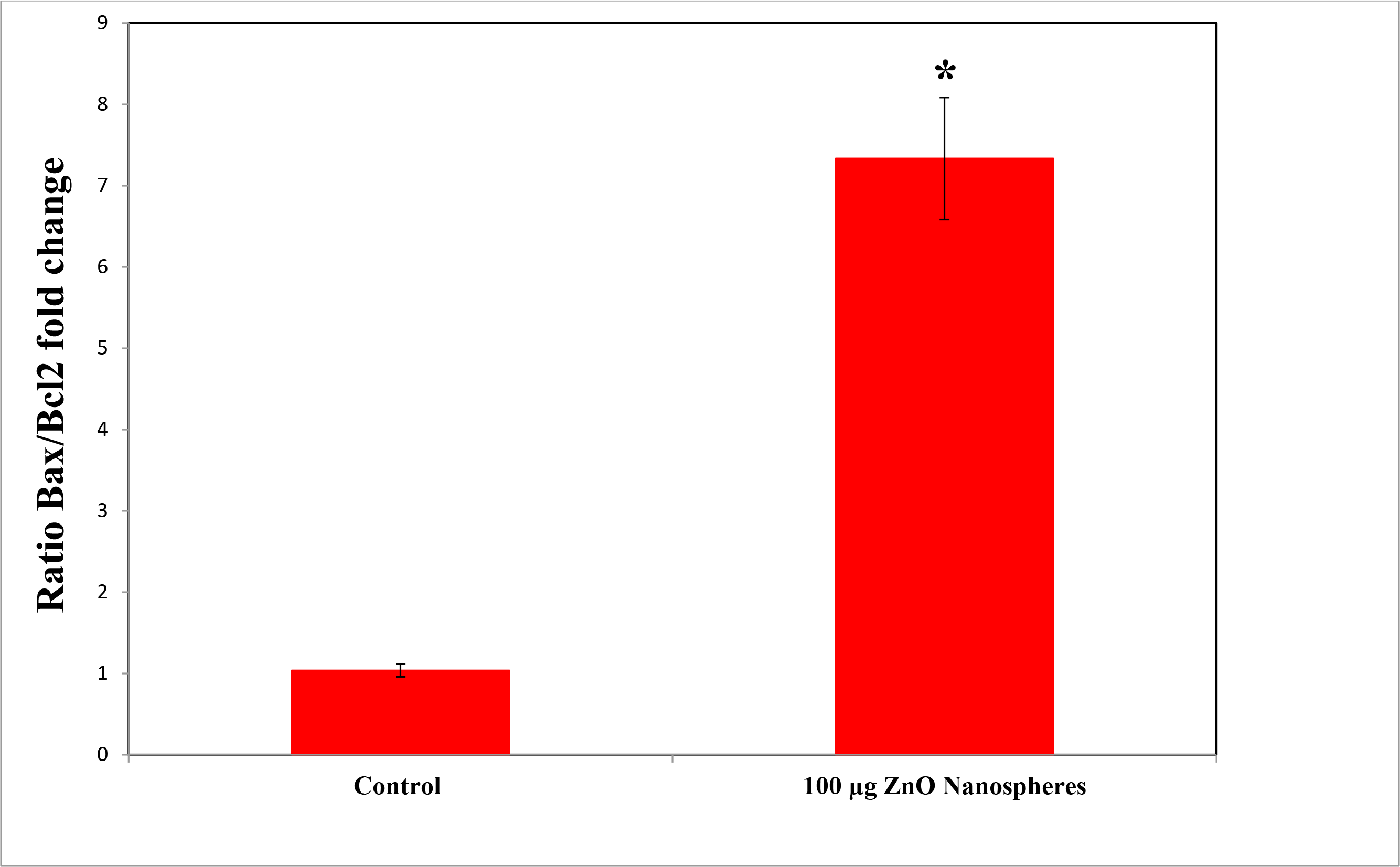

Supplement: Supplemental image S1 — Bax/bcl2 ratio. [file Image_1.tif]
